# Nutritional Benefits of Lycopene and Beta‐Carotene: A Comprehensive Overview

**DOI:** 10.1002/fsn3.4502

**Published:** 2024-10-16

**Authors:** Tabussam Tufail, Huma Bader Ul Ain, Sana Noreen, Ali Ikram, Muhammad Tayyab Arshad, Muhammed Adem Abdullahi

**Affiliations:** ^1^ School of Food & Biological Engineering Jiangsu University Zhenjiang China; ^2^ University Institute of Diet and Nutritional Sciences The University of Lahore Lahore Pakistan; ^3^ University Institute of Food Science and Technology The University of Lahore Lahore Pakistan; ^4^ Department of Food Science and Postharvest Technology Jimma University College of Agriculture and Veterinary Medicine, Jimma University Jimma Ethiopia

**Keywords:** Antioxidant, Beta‐carotene, Carotenoid, Health Benefits, Lycopene

## Abstract

Certain carotene components, such as lycopene and beta‐carotene, are found in tomatoes, carrots, sweet potatoes, etc. and are good for human health. It gives plants their distinctive red color. A class of lipid‐soluble natural pigments known as carotenoids is the precursor of vitamin A and is vital for antioxidant defense against peroxides in cells and tissues. This review provides an overview of the current state of knowledge and research on the sources, structures, physiochemical properties, absorption and metabolism, functional advantages, and prevention of many diseases associated with lycopene and beta‐carotene. These antioxidants have been linked to a lower risk of cardiovascular disease and cancer, and they also help draw pollinators to flowers. Carrots and sweet potatoes are also rich sources of beta‐carotene, which strengthens the immune system and improves eye health. The vivid color of many plants reproductive organs, including flowers and fruits, is caused by carotenoid, a secondary metabolite that is produced in plastids. The distinctive red color and many other health advantages are attributed to lycopene. When ingested through food or supplements, lycopene and beta‐carotene help manage and prevent a number of diseases, including cancer, metabolic, inflammatory, cardiovascular, hepatic, ophthalmic, skeletal, and infertility disorders. The main point is that toxicity is uncommon, and these carotenoids are generally accepted to be safe at different doses. Including these nutrients in your diet can improve your general health and provide illness prevention.

## Graphical abstract

This comprehensive review explores the sources, structures, physiochemical properties, absorption, metabolism, and functional advantages of lycopene and beta‐carotene, crucial carotenoid antioxidants for human health. We discuss their roles in disease prevention, including cardiovascular disease, cancer, and metabolic disorders, as well as their contributions to immune support, eye health, and overall well‐being.

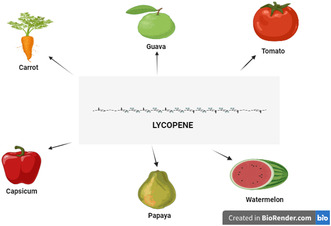

## 1 Introduction

Phytochemicals are bioactive, non‐nutritive compounds produced by plants that display health‐promoting and disease‐preventive attributes upon ingestion. Carotenoids and phenolics are the two main classes of phytochemicals that possess undenied evidence to reduce the risk of numerous diseases (González‐Peña et al. 2023). More than 10,000 phytochemicals have been discovered, and still, an innumerable percentage has to be found. The phytochemicals identified include carotenoids, triterpenoids, tannins, steroids, saponins, alkaloids, and flavones (Sharma and Kaushik 2021). Carotenoids are a group of C40 liposoluble terpenoids that contain over 600 pigments produced by various plants and about 46 microorganisms. These components are stored in plastids of fruits and vegetables that exhibit bright colors of red, orange, and yellow (Xu et al. 2024). Human blood is reported to contain around 40 of these carotenoids (Bohn et al. 2023).

Carotenoids are further classified according to their structure into carotenes and xanthophylls. Carotenes contain only carbon and hydrogen molecules and lack oxygen (Ademowo et al. 2024). They can be linear or cyclic at one or both ends of molecules like lycopene, beta‐carotene, and beta‐cryptoxanthin. On the other hand, xanthophylls contain oxygen and are derivatives of carotenes. Lutein, astaxanthin, and zeaxanthin belong to this class (Elbahnaswy and Elshopakey 2024; Kainat et al. 2022). Lycopene is a lipid‐soluble tetraterpenoid that contains eight isoprene units. Ernest discovered lycopene in 1959. Schnuck named it lycopin, while Escher proposed its chemical structure (Li, Cui, and Hu 2023; Li, Xu, et al. 2023; Li, Zhan, et al. 2023). In today's era, lycopene is a ubiquitous component of human diets all around the globe. Humans cannot synthesize lycopene; therefore, it needs to be taken through diet (Wang, Heng, Song, et al. 2023; Wang, Li, Duan, et al. 2023; Wang, Lin, Liu, et al. 2023; Wang, Shen, Li, et al. 2023; Wang, Xu, Tan, et al. 2023; Wang, Zhang, Yin, et al. 2023; Wang, Zhang, Zhang, et al. 2023; Wang, Zhou, Zheng, et al. 2023). There is a significant disparity in doses of lycopene from individual to individual, but on average, the West consumed 5–7 mg of lycopene per day (Fenech, Bull, and Van Klinken 2023). Lycopene belongs to the carotene family of carotenoids. It is an acyclic polyunsaturated hydrocarbon lacking vitamin A activity (Wal et al. 2023). Lycopene comes under the umbrella of natural pigments. It is responsible for the peculiar red pigment in various fruits and vegetables, mainly tomatoes, watermelon, and pink grapefruit (Gupta et al. 2024). Despite other sources, lycopene is abundant in tomatoes and tomato‐based products. They are reported to provide 80% of total lycopene in the human diet (Landrier et al. 2023).

Plants and microorganisms synthesize lycopene and other carotenoids with the help of photosynthesis. All animals take carotenoids by ingesting plants in their diet. Lycopene is a major serum carotenoid in human blood and tissues. The liver and adipose tissues are the main storage organs for lycopene, in addition to the adrenal and prostate glands. The liver concentration of lycopene is 1.28–25.46 nmol/g, while 0.20–0.70 nmol/g is present in adipose tissues. Human plasma concentration of lycopene ranges from 0.22 to 1.06 nmol/mL and has a half‐life of 2–3 days inside the body (Lilly et al. 2024). Lycopene belongs to predominantly used pigments in the food industry and has wide applications as a food additive and supplement. It is also extensively utilized in cosmetics, food, and beverage industries as a naturally occurring colorant due to its highly liposoluble nature (Di Salvo et al. 2023).

Epidemiological studies reported the efficacy of foods containing high amounts of lycopene in promoting health and well‐being. Due to its distinctive free radical scavenging activities, lycopene confers significant prevention against chronic maladies like cancer and inflammatory, neurodegenerative, metabolic, and skeletal diseases (Crupi et al. 2023). The ability of lycopene to quench free radicals is twice more than that of beta‐carotene and tenfold more than that of α‐tocopherol. Studies also report lycopene's immunomodulating and hormonal modulating activities and its anticarcinogenic activities (Boulaajine and Hajjaj 2024). Beta‐carotene is the most abundant carotenoid in the diet, serum, and human body tissues. It is found in almost all body parts and tissues. Beta‐carotene mainly imparts yellow‐orange color to fruits and vegetables. The prime sources are carrots, mangoes, squash, sweet potatoes, apricots, and dark green leafy vegetables like spinach (Dukat 2023). Like lycopene, beta‐carotene is also a 40‐carbon compound. The presence of 15 double bonds that are conjugated, polar in nature, and 2 beta‐ionic rings at both ends of molecules makes it extremely hydrophobic (Jin and Arroo 2023).

The primary source of vitamin A for humans is through beta‐carotene ingestion. Beta‐ carotene molecule can split and supply two retinol molecules upon the actions of enzyme β‐carotene 15,15′‐monooxygenase. Hence, it is said to have provitamin A activity or a precursor of vitamin A (Ebadi et al. 2023). The provitamin A activity of beta‐carotene is more than any other carotenoid, and the deficiency of beta‐carotene in diet can result in conditions like blindness, dryness of eyes, and premature infant death (Farasati Far et al. 2023). Adipose tissues act as the main storage organs for beta‐carotene, containing 80%–85% of the total beta‐carotene in the human body. The liver and muscles store the rest in concentrations of 8%–12% and 2%–3%, respectively. Hence, studies concluded that the liver and adipose tissues are the major storage houses of beta‐carotene (Choi and Yun 2023). Up to 45% of beta‐carotene taken through diet remains intact in the animal body, thus proposing it is highly absorbable, utilizable, and bioavailable. Disease protective role of beta‐carotene against ophthalmic diseases, metabolic diseases, and cardiovascular diseases has been proven by research studies (Bakac et al. 2023).

Due to the provitamin A activity of beta‐carotene, the human body utilizes it for growth, embryonic development, and maintaining eyesight. Numerous health benefits make it an important medical component (Kurniawan, Nora, and Sari 2023). Beta‐carotene also exhibits anticarcinogenic, gene‐modulating, and free radical scavenging properties. Beta‐carotene is an important food additive due to its functional properties. Furthermore, it is also utilized as a natural pigment in various industries. To manifest yellow to orange pigment as a colorant in foods, beta‐carotene is used in the concentration of 2–50 ppm. Provitamin A's potential makes it an important nutritional supplement (Nabi et al. 2023).

## 2 Sources

**FIGURE 1:**
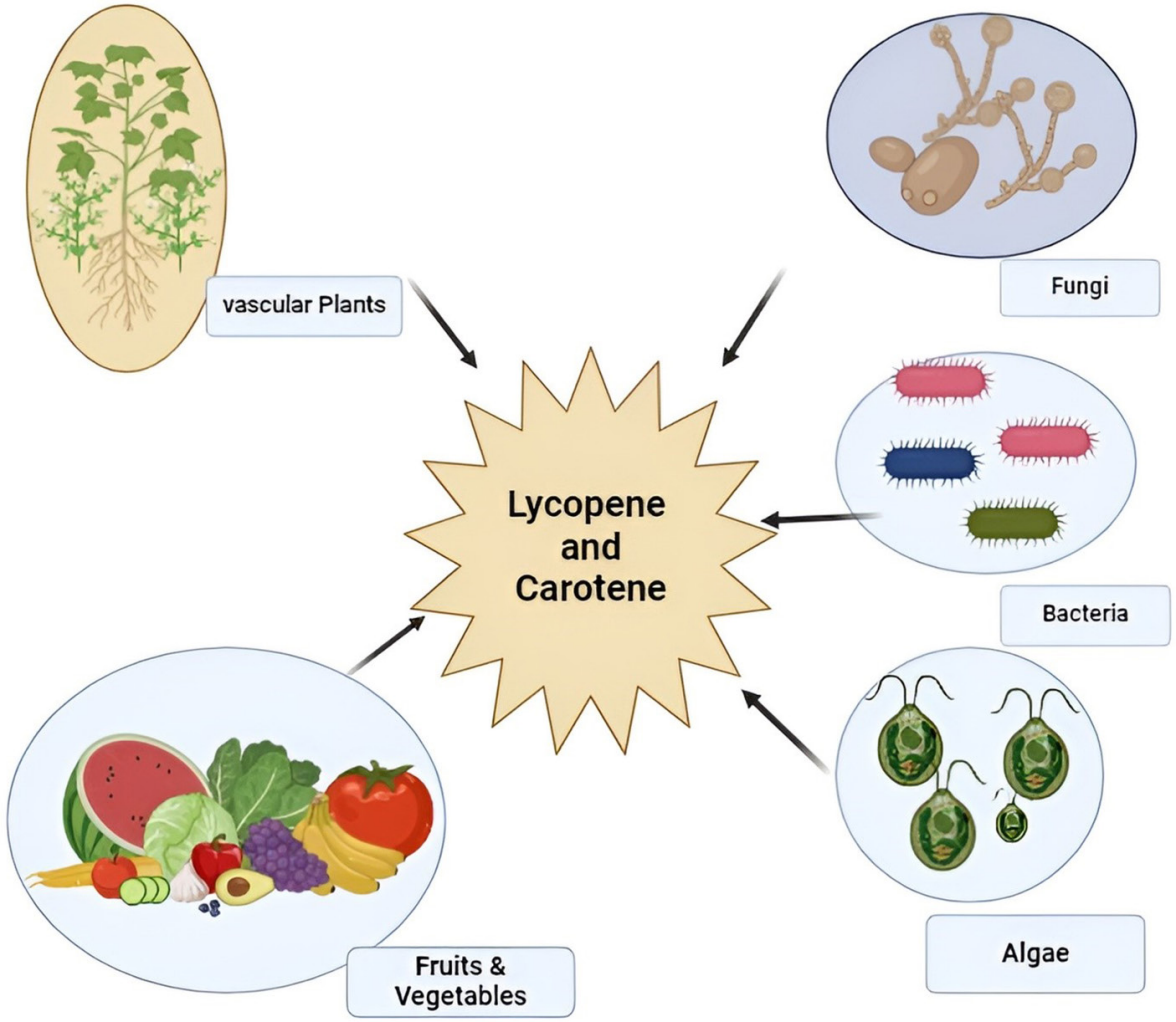
Main sources of lycopene.

The prime sources of lycopene are vascular plants and microorganisms like bacteria, algae, and fungi. All animal kingdoms, including humans, cannot synthesize lycopene and must consume it through plants. Another microorganism Marine Haloarchaea belongs to the Haloferacaceae family and synthesizes lycopene (Ashokkumar et al. 2023). Reddish fruits and vegetables like tomatoes, watermelon, apricots, guava, carrots, capsicum, and papaya are rich sources of lycopene, as shown in Figure 1. Tomato‐based products like ketchup, tomato paste, soups, and sauces also contain significant amounts of lycopene (Landrier et al. 2023). Lycopene is responsible for the pigmentation of plants' fruits, flowers, and stems as it binds with fiber due to its liposoluble nature. The commercial production of lycopene and beta‐carotene for additives and supplements is obtained from a fungus called Blakeslea transport, also known as a fungal plant pathogen. Industrial and laboratory production of lycopene is performed to make supplements in capsules, gel, or tablet forms (Olaniran et al. 2023).

Tomatoes are the cheapest raw material for extracting lycopene, providing 80% of the total lycopene used in various industries. Tomato lycopene is present as crystalline long needles, which can precipitate in the aqueous environment (Yin et al. 2023). Processing causes significant loss of water, thus reducing lycopene concentration in processed tomato‐based products. Fresh tomatoes contain much more lycopene than these processed products (Baghabrishami and Goli 2023).

The maturity of fresh fruits, the family they belong to, agricultural land conditions, and climatic and environmental situations impact lycopene content, thus exhibiting variation in different plants (Pathak and Sagar 2023). Red watermelon contains 40% more lycopene content than tomato lycopene. Red melon contains up to 7.2 mg/100 g, while fresh tomatoes contain up to 4.20 mg/100 g. Yellow and yellow‐orange colored contain relatively less lycopene than red ones (Doan et al. 2023). Table 1 shows lycopene content in various foods. Beta‐carotene is significantly present in orange‐red colored plants like carrots, grapefruits, apricots, paprika, cabbage, asparagus, and green leafy vegetables like broccoli and spinach. Chief vegetable sources of beta‐carotene include green chilies, yellow pumpkins, green beans, fresh beans, and pursley. Tomatoes are rich in lycopene but contain beta‐carotene in low amounts (Singh et al. 2023). Cereals provide very low lycopene concentration, while pulses are a relatively better source. Red gram is a very rich source of beta‐carotene. Grains like maize, rice, wheat, sorghum, and millet contain beta‐carotene in varying concentrations (Sarkar et al. 2023). Peppers contain a lot of beta‐carotene, a pigment that gives plants vibrant colors. The concentration of beta‐carotene, which the body converts into vitamin A, rises with the depth and intensity of the pepper's color (Ponder, Kulik, and Hallmann 2021). Peppers are a nutrient‐dense and beneficial food choice since they are high in vitamins, minerals, and fiber while low in calories. Including various peppers in your diet will increase your nutrient intake overall. Including peppers in your meals effectively increases your beta‐carotene intake while encouraging improved health and disease prevention (Arimboor et al. 2015; Khan et al. 2021).

**TABLE 1:** Lycopene content of foods (Radtke et al. 2023).

Food content	(mg/100 g)
Fresh tomatoes	0.88–4.20
Sun‐dried tomatoes	45.90
Cooked tomatoes	3.70
Tomato sauce	6.20
Tomato paste	5.40–150.00
Tomato juice	9.04
Ketchup	9.90–13.44
Dried apricot	0.86
Pink grapefruit	3.36
Guava	5.40
Watermelon	2.30–7.20
Papaya	2.00–5.30
Red carrots	6.1
Pitanga	7.3
Autumn olive	15–54
Bitter melon	41
Gac fruit	34.8
Rose hips	2.2

Fruits that contain beta‐carotene in rich concentrations are mango, bananas, lychee, pear, papayas, pineapple, guava, orange, and watermelon. Spices and herbs like nutmeg, red chili, and fenugreek seeds have significant quantities of beta‐carotene (Yahia et al. 2023). Spices are reported to contain significant carotenoid content, especially beta‐carotene. Nutmeg is the richest source of spices. Clove and turmeric are poor sources (Ahmad, Ibrahim, and Abdelhamid 2023; Ahmad, Khan, et al. 2023; Ahmad, Riaz, et al. 2023). Table 2 displays the reported concentration of beta‐carotene in various plant sources. Microorganisms like fungi, bacteria, algae, and yeasts are used in biotechnology to produce beta‐carotene. Industries use various extraction procedures to extract natural beta‐carotene from plants. Ninety‐eight percent of beta‐carotene is synthesized, while only 2% is naturally produced (Ebadi et al. 2023).

**TABLE 2:** Beta‐carotene content in various food sources.

Source	Content	Reference
Carrot	11,210 (μg/100 g)	
Tomato	3500 (μg/100 g)	
Spinach	9940 (μg/100 g)	Ahamad et al. (2007)
Lady's finger	3220 (μg/100 g)	
Brinjal	2100 (μg/100 g)	
Green chilli	1750 (μg/100 g)	Ahamad et al. (2007)
Strawberry	8.5 (μg/100 g)	
Green beans	0.23 (μg/g)	
Grapes	6.6 (μg/100 g)	Charoensiri et al. (2009)

## 3 Intake and Safety

Due to dietary variations, different regions consume lycopene in various amounts. Dietary surveys conducted by EFSA concluded that different populations in the same country showed variability in lycopene intake. European's lycopene intake was around 0.5–5 mg/day (Gebregziabher et al. 2023). French, British, Dutch, and Irish populations consume more lycopene (up to 5 mg/day) than Spaniards, whose intake was < 1.64 mg/day. Italian's daily tomato‐based diet was 175 g, the Spanish consumed up to 65 g, and the British consumed only up to 30 g (Zamani et al. 2023). Various human and animal research studies have been conducted to evaluate the safety and toxicity of lycopene at different doses. These studies aimed to assess its toxic effects on genes, the liver, the reproductive system, and overall metabolism (Aboubakr et al. 2023). No upper limit of lycopene has been reported, and it is said to be safe and tolerated at varying concentrations, both in diet and supplements. Lycopenemia was the only condition reported due to exorbitant lycopene intake via consuming up to 2 L of tomato juice (Macar et al. 2023). Previously, research studies recommended up to 30 mg of lycopene intake. Advanced research data indicates a daily intake of 0.5–10 mg of lycopene, which can be increased to 20 mg if the consumption of tomatoes and other plant sources of lycopene is high (Zamani et al. 2023). The daily intake of 3 g of lycopene per kg of body weight is safe and does not cause any toxic effects at this concentration (Mirahmadi, Aghasizadeh, Esmaily, et al. 2023; Mirahmadi, Aghasizadeh, Nazifkar, et al. 2023).

Beta‐carotene is a precursor of vitamin A and is converted to vitamin A in the intestine. It is considered to be a safe source of this vitamin. It does not cause toxicity in the body because the high concentration of beta‐carotene in the diet slows the intestinal conversion rate (Farasati Far et al. 2023). Intake of natural beta‐carotene from the diet is considered safe even at large doses and does not show any toxic effects. Supplemental beta‐carotene can exhibit toxicity at higher concentrations, leading to unwanted side effects and disturbances in the human body. Americans and British are reported to consume up to 2 mg of beta‐carotene from dietary sources, while vegan and vegetarian consumption was up to 9 mg/day. Beta‐carotene intake can also be measured in terms of retinol equivalency. The daily dose of 1.5 mg of beta‐carotene equalizes 2500 IU of vitamin A or 250 retinol equivalents. Both lycopene and beta‐carotene have been generally recognized as safe (GRAS) (Watcharawipas, Kocharin, and Runguphan 2023).

## 4 Applications

Carotenoids are widely used in various industries as a colorant, additive, or functional element. Lycopene and beta‐carotene are the most utilized carotenoids. These carotenoids are highly unstable due to oxidation and isomerization, so microencapsulation increases their stability (Li, Cui, and Hu 2023; Li, Xu, et al. 2023; Li, Zhan, et al. 2023). Due to its functional properties and potential in medicine, lycopene is used in pharmaceutical formulations to yield supplements as a tablet or capsule. It is usually combined with a vitamin or mineral supplement (Wei et al. 2023). Functional properties also increase its utilization and highlight its importance as an antioxidant additive or coloring agent in the food industry, pharmaceuticals, and cosmetics. Synthetic lycopene is more commonly used in industries than natural ones. Lycopene supplements soft gel capsules and lycopene drinks (Li, Cui, and Hu 2023; Li, Xu, et al. 2023; Li, Zhan, et al. 2023). In the meat industry, it is used in the form of powder or dried tomato peel to incorporate in various meat products to increase nutritional value, modify texture, as a colorant, as a preservative to protect from rancidity, and as a flavor enhancer of the final product (Dahab et al. 2023). Lycopene is incorporated into a variety of foods to increase its value. Baked products, beverages, juices, cereals, ice creams, jams, jellies, confectionery, and salad dressings are common foods incorporated with lycopene (Li, Cui, and Hu 2023; Li, Xu, et al. 2023; Li, Zhan, et al. 2023).

Previously, synthetic colorants have been reported to deteriorate health by causing unwanted side effects. Researchers then emphasized the importance of using natural coloring agents such as lycopene and beta‐carotene that provide pigment and can add value to the food, thus improving overall health. For this purpose, these carotenoids are extracted using different procedures for further use in industries (Di Salvo et al. 2023). Beta‐carotene imparts the color yellow to orange when added to food products. It is usually used as a colorant in a 2–50 ppm concentration. Due to its vitamin A activity, beta‐carotene is widely used as a supplement and is easily available (Hari et al. 2023). Vitamin A deficiency is quite prevalent in the world, especially in children. To combat nutritional deficiency, staple or regularly used food products are biofortified. Foods like maize and sweet potatoes are commonly fortified with beta‐carotene using bioengineering to enhance their health value.

Beta‐carotene is unstable when added to foods as it is highly prone to thermal, light, and oxidative degradation. Various processing techniques are being applied to increase the shelf‐life of various food products. As a coloring agent, beta‐carotene is added to foods such as ice creams, confectionary, baked products, fruity beverages, and fats to impart orange‐red pigment. The pharmaceutical industry uses it as a natural pigment in tablets, capsules, and syrups (Huang, Liu, and Pan 2023). Due to its antioxidant potential, beta‐carotene is utilized as a bioactive ingredient in multiple formulations in the cosmetic industry to prevent skin oxidation, ultraviolet radiation, and, thus, skin cancers. It is also a natural pigment in cosmetics (Suryana et al. 2023).

Beta‐carotene offers health benefits other than vitamin A activity. It is an effective free radical scavenger that helps boost immunity and prevent and combat antioxidant stress, cancer, metabolic disease, cardiovascular diseases, and ophthalmic diseases. Numerous human trials have documented the protective and health‐boosting potentials of both lycopene and beta‐carotene when taken as a supplement or from carotenoid‐rich food sources (Lampousi et al. 2024).

## 5 Bioavailability

The bioavailability of carotenoids is dependent on multiple factors. Lycopene's bioavailability is affected by cooking, cutting/chopping of food, dose, interaction with other carotenoids, presence of fats/oils, and physiological condition of the person ingesting it (Cakir and Helvacioglu 2023). In advanced age, the genetic characteristics of individuals and the presence of dietary fibers can reduce fat and lycopene absorption in the intestines. All of the factors mentioned above influence the detachment of lycopene from the food matrix, digestion, and absorption (Wang, Heng, Song, et al. 2023; Wang, Li, Duan, et al. 2023; Wang, Lin, Liu, et al. 2023; Wang, Shen, Li, et al. 2023; Wang, Xu, Tan, et al. 2023; Wang, Zhang, Yin, et al. 2023; Wang, Zhang, Zhang, et al. 2023; Wang, Zhou, Zheng, et al. 2023). During cooking and processing, the bioavailability of lycopene is increased due to copping and pureeing as it lessens the fragment size. Lycopene is a hydrophobic and somewhat lipophilic compound. The presence of water reduces its solubility and bioavailability (Li, Cui, and Hu 2023; Li, Xu, et al. 2023; Li, Zhan, et al. 2023). The presence of lipids and fats makes it relatively more soluble and bioavailable than water, but generally, the solubility of lycopene in fat is not that great. Lycopene is reported to be only 23% soluble when mixed with oil and up to 5% when consumed with tomato juice or products. This shows low bioavailability (Baghabrishami and Goli 2023).

The presence of fats in meals helps absorb carotenoids to some extent. This finding has increased researcher's interest in studying the interrelation of lipids with lycopene. When ingested animal, plant‐based, and dairy fats may help absorb and enhance lycopene bioavailability (Wu, Wu, et al. 2023; Wu, Zhu, et al. 2023). Geng et al. (2023) conducted a study on the effect of various lipids in varying amounts on the digestion and extent of lycopene absorption. They concluded that its absorption is affected by the amount of ingested fat and not by the type or source of fat. Incorporating oil in carrot products containing lycopene reduces its stability in the end product. Lycopene stabilizes tomatoes and tomato‐based products during heat processing and storage (Hanjabam et al. 2024). Processing techniques also help break down the cell wall of plants, causing a significant reduction in the adhesion of lycopene with tissue matrix. This leads to more accessibility of lycopene for digestive enzymes that convert it to cis‐isomer and enhance bioavailability (Zahari et al. 2023). Lycopene is naturally present as a trans‐isomer in fruits and vegetables. This form is poorly absorbed and significantly less bioavailable. Thermal processing of tomato‐based products like ketchup, pastes, etc., causes a lycopene structure shift and yields cis‐isomer in the product. This form of lycopene is more bioavailable. Cis‐isomers show greater solubility and absorbance in the human gastrointestinal tract (GIT) than trans isomers (Rawat, Siddiqui, and Singh 2023).

A study by Ennab et al. (2023) revealed that calcium consumption with lycopene affects its bioavailability. Calcium forms chelated with fats and forms insoluble complex structures that reduce the bioavailability by 84%. Lycopene interacts with molecules present in food, like fatty acids and sulfur. These molecules induce a change in the isomerization of lycopene into cis form and, thus, enhance absorption and bioavailability. Beta‐carotene is present as crystalline formation in the plastids of plant sources like fruits and vegetables. It makes photosynthetic complex structures with compounds in these cells. Absorption of beta‐carotene in GIT is influenced by numerous factors such as host‐related factors (genetic makeup/nutritional status/physiological condition and their interaction), the concentration of consumed beta‐carotene, conversion into vitamin A, nature of the meal, presence of other food components like lipids, rate of digestion, absorption, and metabolism inside the body (Butnariu 2023). Various factors also influence the bioavailability of beta‐carotene. Other food compounds have been reported to diminish the bioavailability of beta‐carotene, but each component exerts a different effect (Kaeppler, M. S. 2023). The dietary source of beta‐carotene and adherence to the matrix influence the absorption and bioavailability significantly. The bioavailability of lycopene directly absorbed from vegetable sources is only up to 14%, while extracted, simplified, and purified beta‐carotene shows more bioavailability (Niaz and Mackie 2024). In natural sources, beta‐carotene is present as a crystalline structure, which makes absorption hard. It also forms insoluble complexes with proteins that interfere with the digestive processes of GIT by impairing release (Muthuraman et al. 2023).

## 6 Structure and Chemical Properties

Lycopene belongs to the tetraterpenoid class of carotenoids, consisting of eight isoprene units held together by tail‐to‐head configuration. The central structure is joined by a tail‐to‐tail configuration, which aligns the structure with symmetry. Other names for lycopene are trans lycopene and γ,γ‐carotene (Wal et al. 2023). The distinguishing characteristic of lycopene is the presence of double bonds along the length of the molecule. They help absorb light in the visible range in plant cells and exhibit vibrant pigment used as a colorant (Gupta et al. 2024).

**FIGURE 2:**
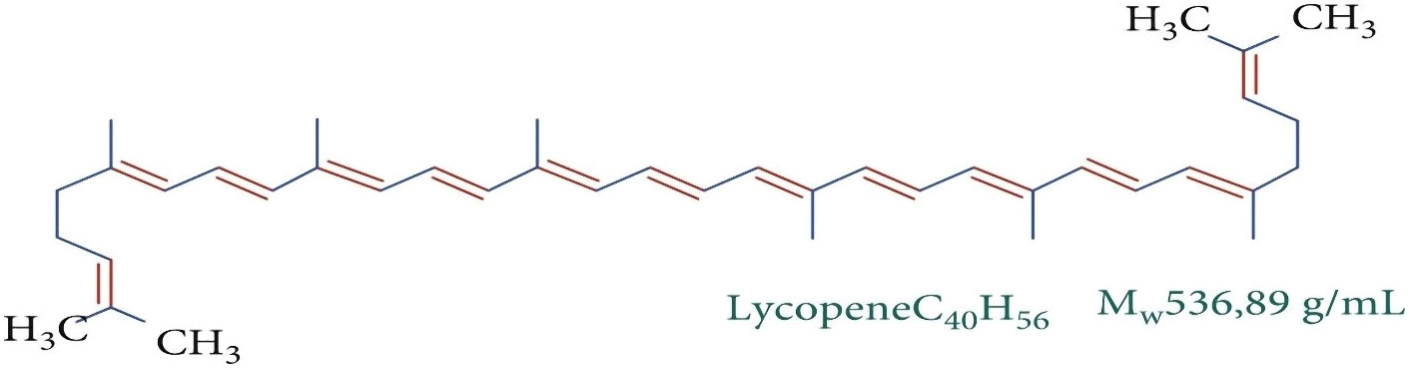
Chemical structure of lycopene (Khan et al. 2021).

**FIGURE 3:**
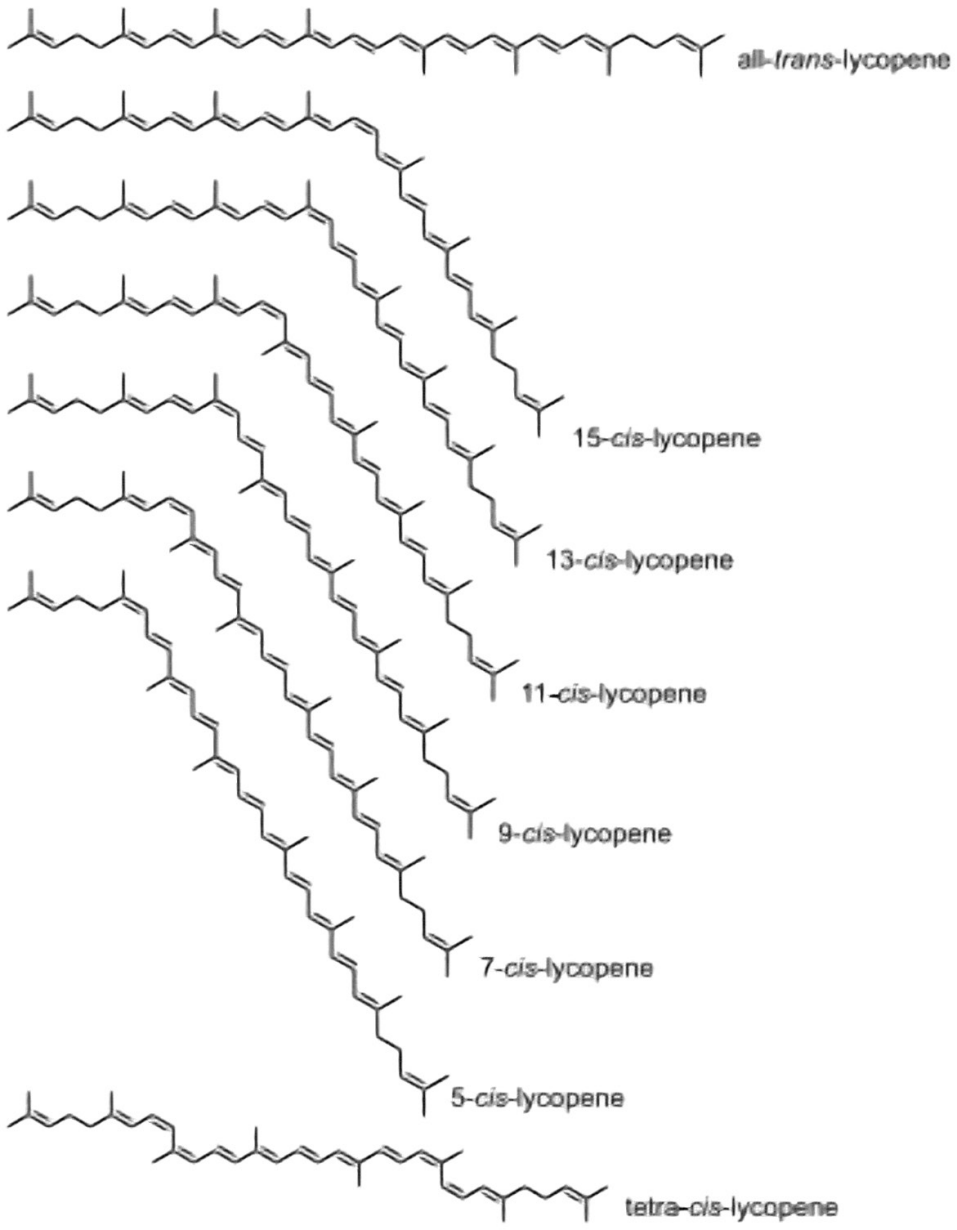
Molecular structures of lycopene isomers (Arballo et al. 2021).

Dewi and Susilo (2023) presented the chemical structure of lycopene by categorizing its breakdown products. Lycopene is known as 2, 6, 10, 14, 19, 23, 27, 31—octamethyl—2, 6, 8, 10, 12, 14, 16, 18, 20, 22, 24, 26, 30—dotriacontatridecaene in scientific nomenclature. The chemical and molecular structure of lycopene are shown in Figures 2 and 3, respectively.

The presence of double bonds favors the existence of lycopene in both cis and trans isomers. Naturally, lycopene exists as all‐trans lycopene as it is highly stable. Lycopene has multiple cis‐isomers, such as 5‐cis, 9‐cis, 13‐cis, or 15‐cis, which exhibit relatively greater solubility. Lycopene is famous for attaining up to 2048 structural configuration (Nunes et al. 2023).

Interaction with light, thermal energy, chemicals, and other food components changes lycopene's configuration into mono Z or poly Z cis form by isomerization procedure. In human organs and blood plasma, lycopene forms mixtures of isomeric structures in which 65% lycopene is present as any Z form while the remaining lycopene is present as E forms (Liu, Lin, et al. 2023; Liu, Zhou, et al. 2023). Lycopene does not have a ring, meaning it is acyclic and is in liner form with 11 conjugated and two unconjugated double bonds. It is known as an open‐chain derivative of beta‐carotene. The stability of various configurations is different. The 5‐cis form has the highest stability and antioxidant activity (Tripathi et al. 2024). The distinctive ruby‐red pigment of lycopene is due to multiple conjugated double bonds. It also accounts for free‐radical scavenging activity. Lycopene cannot act as a provitamin A compound, unlike other carotenes, such as alpha/beta‐carotene. The absence of cyclic configuration and beta‐ring is responsible for this (Baç, Yemiş, and Özkan 2023). In blood plasma, lycopene is a mixture of its configurations with cis‐form in 40%–50%. Lycopene is the principal carotenoid of blood, making up 43% of the total content. Since lycopene is only soluble in lipids and fats, it is bound to low‐density (LDL) and very low‐density lipoproteins (VLDL) in blood plasma (Abir et al. 2023; Noreen et al. 2023).

**FIGURE 4:**
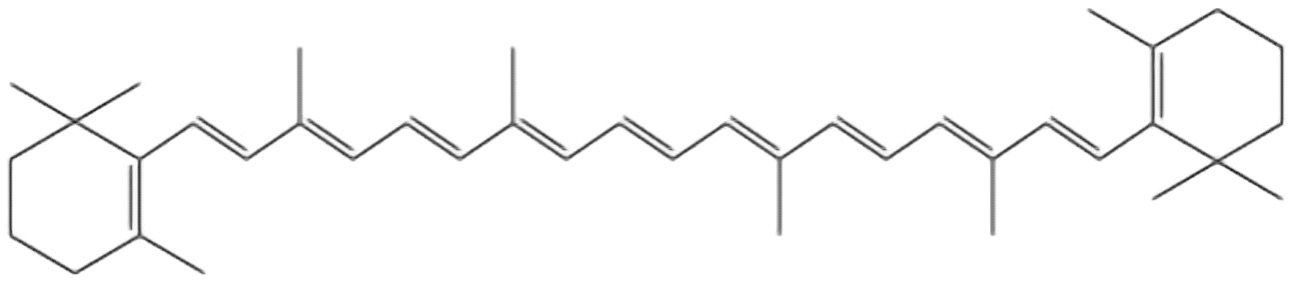
Chemical structure of beta‐carotene (Wingqvist 2011).

According to Bradley et al. (2023), there is a variation in the half‐life of lycopene in blood. Some researchers say it is 2–3 days, while other studies have reported 12–33 days. Higher plants synthesize beta‐carotene as a secondary product, which does not oxidize. It is derived from the acyclic configuration that contains multiple double bonds in a liner structure. Exposure to thermal energy causes isomerization of these double bonds, leading to the production of vibrant color (Wang, Heng, Song, et al. 2023; Wang, Li, Duan, et al. 2023; Wang, Lin, Liu, et al. 2023; Wang, Shen, Li, et al. 2023; Wang, Xu, Tan, et al. 2023; Wang, Zhang, Yin, et al. 2023; Wang, Zhang, Zhang, et al. 2023; Wang, Zhou, Zheng, et al. 2023). More than 600 liposoluble carotenoids exist in nature and are synthesized by various plants and microorganisms. Beta‐carotene belongs to this class with an isoprenoid structure having two C‐20 geranyl‐geranyl diphosphate molecules joined together by tails, which leads to the formation of C‐40 molecules (Wal et al. 2023). Beta‐carotene has two beta‐ionic rings in its structure that are responsible for provitamin A activity. Breaking the linear chain at C15 and C15's double bond location yields two retinol molecules (Nurhasanah and Munarso 2024). The chemical structure of lycopene is shown in Figure 4. C40 acts as a parent structure for further derivations. Beta‐carotene is a mixture of trans and cis forms, with all trans‐forms being dominant. Cis‐form is also present but in different concentrations, depending on the food source. The order of concentration of different configurations is all‐trans > 9‐cis‐ > 13‐cis > 15‐cis (Żbik, Kłodawska, and Malec 2023).

Beta‐carotene in transform shows instability, and isomerization into cis‐form makes it stable. Exposure to high heat, light, chemicals, and oxygen during processing techniques and storage can oxidize and change trans beta‐carotene into cis‐isomer. Thermal energy enhances the isomerization process, thus enhancing the vibrancy of the pigment provided by beta‐carotene (Sanpapao et al. 2023).

## 7 Physical Properties

Lycopene is a polyunsaturated organic compound with only carbon and hydrogen in its linear structure. The molecule of lycopene weighs around 536.85 Da and has the formula C40H56. It shows thermal, oxidation, chemical, and photo‐sensitivity. The melting point of lycopene is up to 175°C (Sheikh et al. 2023). Crystalline formulation of lycopene exists as red elongated needles that contain carbon disulfide and ethanol in their structure. Powder form exhibits deep reddish‐brownish pigment. Double bonds increase solubility in organic solvents such as chloroform, benzene, acetone, etc. (Wei et al. 2023). The molecular formula of beta‐carotene is C40H55 with weight of 536.88 Da. Like lycopene, beta carotene also exists as either powdered or crystalline, exhibiting pigments ranging from red to deep red. Beta carotene is also liposoluble and hydrophobic (Li, Cui, and Hu 2023; Li, Xu, et al. 2023; Li, Zhan, et al. 2023).

## 8 Synthesis

Plants synthesize pigments like lycopene when they absorb light while performing photosynthesis. Lycopene is liposoluble and exhibits a red color. Synthesis and concentration of lycopene in various plants depend on the plant's genetic factors and environmental conditions (Wang, Heng, Song, et al. 2023; Wang, Li, Duan, et al. 2023; Wang, Lin, Liu, et al. 2023; Wang, Shen, Li, et al. 2023; Wang, Xu, Tan, et al. 2023; Wang, Zhang, Yin, et al. 2023; Wang, Zhang, Zhang, et al. 2023; Wang, Zhou, Zheng, et al. 2023). The chloroplast is the plastid responsible for photosynthesis. It contains chlorophyll, a pigment that is crucial during photosynthesis. It also provides the leaves with their green color. Chloroplasts specialize in photosynthesis and are distinguished by the presence of an internal thylakoid membrane system. It collects and transports light energy required for glucose synthesis, essentially acting as a catalyst. Leucoplasts synthesize and store carbohydrates, lipids, and proteins. These various plastid types collaborate to promote the growth and development of plant cells and tissues (Dobrogojski, Adamiec, and Luciński 2020; Rolland et al. 2018). This leucoplast further leads to the formation of red‐pigmented chromoplast plastids. Acetyl coenzyme A, via the isoprenoid pathway, formulates an isoprenoid precursor, isopentenyl diphosphate (IPP), a five‐carbon compound. It is then converted to geranyl pyrophosphate (GPP) (Jayarathna, Navaratne, and Wickramasinghe 2023). In the later stage, two IPP molecules are added to GPP to convert to GGPP, that is, geranylgeranyl pyrophosphate containing 20 carbons. Afterward, two molecules of GGPP are joined together at the position of their heads, removal of water takes place with the help of the enzyme phytoene desaturase, and a new compound containing 40 carbon is formed, which is also known as phytoene (Brychkova et al. 2023).

The plastids in plants produce carotenoids, which are C40 isoprenoids (Dobrogojski, Adamiec, and Luciński 2020; Rolland et al. 2018). To form carotenoids, the universal C5 isoprenoid precursors isopentenyl diphosphate (IPP) and dimethylallyl diphosphate (DMAPP) condense. Plants generate these precursors via two distinct pathways: the mevalonate (MVA) process and the methylerythritol 4‐phosphate (MEP) pathway. The MEP system produces both IPP and DMAPP in the plastid from pyruvate and glyceraldehyde 3‐phosphate. In contrast, the MVA pathway generates IPP in the cytosol from three molecules of acetyl‐CoA and subsequently isomerizes it to DMAPP (Rodríguez‐Concepción and Boronat 2015).

In plastids, precursors generated from MEP are converted into plant carotenoids. The MEP route may have a limiting function in carotenoid production, according to several lines of evidence (Dobrogojski, Adamiec, and Luciński 2020). Deoxyxylulose 5‐phosphate synthase (DXS), the first enzyme in the MEP route, has the highest flow control coefficient, according to metabolic flux assessments (Wright et al. 2014). At several levels, DXS activity is highly controlled (Hemmerlin 2013). In addition to the main control that stems from the regulation of gene expression, plastids also employ a fine control that is mediated by the modulation of enzyme levels. DXS is prone to misfolding and aggregation within plastids. Under typical growth conditions, the stromal Clp protease complex delivers non‐functional versions of the enzyme for destruction once they are identified by a particular J‐protein (Flores‐Perez et al. 2008; Pulido et al. 2013). However, to reactivate the enzyme under stress, a refolding process is initiated (Pulido et al. 2016; Llamas, Pulido, and Rodriguez‐Concepcion 2017). A feedback mechanism involving DMAPP and IPP can also be used to inhibit DXS activity (Pokhilko et al. 2015). Clp protease and redox signal both regulate other MEP pathway enzymes by interacting directly with thioredoxin (Flores‐Perez et al. 2008; Sauret‐Gueto et al. 2006).

**FIGURE 5:**
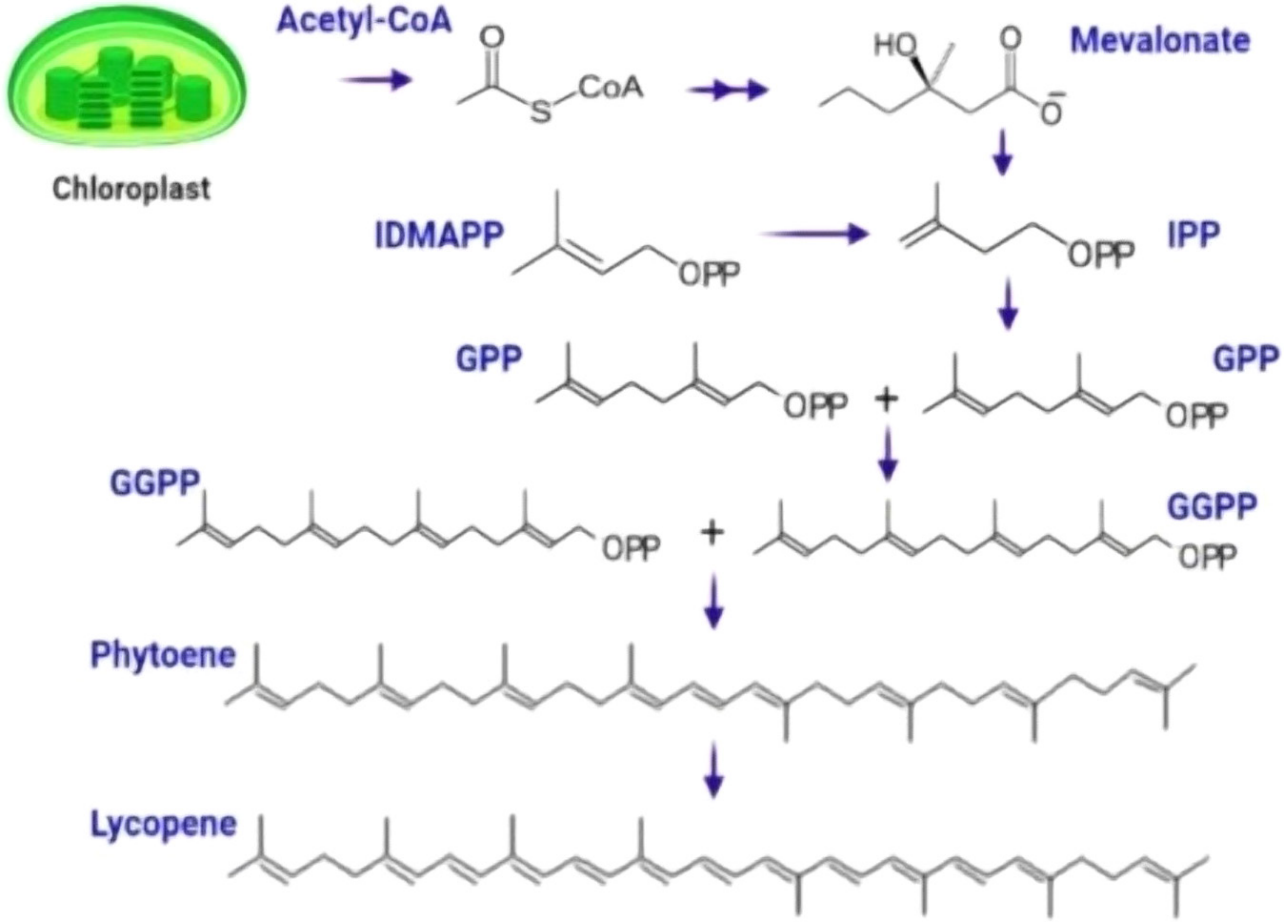
Biosynthesis of lycopene.

Geranylgeranyl diphosphate (GGPP), the direct metabolic precursor of several other plastidial isoprenoids that play important roles in growth regulation (gibberellins), photosynthesis (chlorophylls, tocopherols, plastoquinones, and phylloquinones), and environmental interactions (diterpenes), is formed by the condensation of three IPP and one DMAPP molecules. As a result, numerous metabolic pathways compete for the GGPP that the plastids provide (Ruiz‐Sola, Barja, et al. 2016; Ruiz‐Sola, Coman, et al. 2016). Physical interactions take place between isoforms of plastidial GGPP synthase (GGPPS) and the enzymes that use GGPP to create downstream products such as carotenoids. However, overexpression of GGPP‐producing enzymes from various sources has had no impact on plant carotenoid levels (Zhou et al. 2017; Tata et al. 2016). Since lycopene is soluble in a lipidic environment, it forms a composite with protein in membrane compartments of thylakoid and is stored there (Ma et al. 2023). The synthesis pathway of lycopene is shown in Figure 5.

Lycopene is utilized as a substrate for the formation of other carotenoids. Cyclase enzyme catalyzes the addition of cyclic structures in trans‐lycopene, leading to various carotenoid production. These carotenoids are discriminated based on cycles added. It can be a beta ring or epsilon ring, also known as ε‐ring (Wang, Heng, Song, et al. 2023; Wang, Li, Duan, et al. 2023; Wang, Lin, Liu, et al. 2023; Wang, Shen, Li, et al. 2023; Wang, Xu, Tan, et al. 2023; Wang, Zhang, Yin, et al. 2023; Wang, Zhang, Zhang, et al. 2023; Wang, Zhou, Zheng, et al. 2023). Enzymes such as beta‐cyclase and ε‐cyclase help form these rings, respectively. Beta‐carotene is formed by a similar procedure when two beta‐rings are added to lycopene (da Silva, de Paiva, and Mariutti 2023). Another procedure is used to synthesize lycopene, known as chemical synthesis. It is a three‐phase procedure. At first, a 15‐carbon compound is formulated and diffused into an organic solvent such as methanol. In the next step, the Witting reaction forms a 10‐carbon crystal compound (Wang, Heng, Song, et al. 2023; Wang, Li, Duan, et al. 2023; Wang, Lin, Liu, et al. 2023; Wang, Shen, Li, et al. 2023; Wang, Xu, Tan, et al. 2023; Wang, Zhang, Yin, et al. 2023; Wang, Zhang, Zhang, et al. 2023; Wang, Zhou, Zheng, et al. 2023). In the last stage, thermal energy and enzymes combine these intermediates, yielding lycopene. After cooling, the raw compound is passed through filtration and washing with aqueous and organic solvents. Methanol is then used to purify the crude‐filtered product, and this purified compound is dried using nitrogen (Zhang, Chen, et al. 2023; Zhang, Zhai, et al. 2023). Beta‐carotene is synthesized from lycopene in plants, as mentioned above. Chemically, beta‐carotene is formulated with the help of Grignard reactions. Grignard reagents are used to merge a dicarbonyl molecule with two methanol molecules. Beta‐carotene, a 40‐carbon compound, is obtained as a result (Ebadi et al. 2023). Other procedures used to synthesize beta‐carotene include water removal, dehydration, condensation, and elimination reactions of carbonyl molecules. Homodimers and cross‐coupling of Csp2 molecules are also widely used for beta‐carotene synthesis (Pouzet et al. 2023).

## 9 Extraction Techniques

The extraction of lycopene has taken place since the middle of the 19th century. Various methods of carotenoid extraction are reported in research. Plants and microorganisms are the primary sources of natural lycopene extraction. Customers prefer natural lycopene over artificially synthesized lycopene (Hatami and Ciftci 2023). Some safe techniques used to extract lycopene are enzymatic extraction, supercritical carbon dioxide extraction, microwave and ultrasound‐assisted extractions, etc. Due to their reported safety, researchers are trying to use solvents like methoxymethane for extraction procedures. Extraction of lycopene is influenced by its solubility and stability under various conditions (Kumar et al. 2023). Tomatoes and watermelon are preferred for lycopene extraction. Watermelon pulp contains more lycopene in cis configuration than found in tomatoes. Lycopene shows stability against heat when present naturally in chromoplasts as an all‐trans configurated crystalline form. It is thus regarded as thermoresistant. Traditionally, thermal energy is required to remove the pigment by transporting the solvent (Zahari et al. 2023).

The following are some techniques used to extract carotenoids: lycopene and beta‐carotene.

### 9.1 Solvent Extraction

Organic solvents are widely and frequently used in industries that extract food. This is a trusted and efficient extraction method, but it is arduous and complex and demands proper waste disposal (Viñas‐Ospino et al. 2023). Lycopene is highly soluble in solvents like dichloromethane, trichloromethane, and benzene. These are organic and chemically produced solvents in industries that aid in chemical extraction procedures. Solvent extraction is not much preferred owing to the toxicity of used chemicals. These chemicals are undetectable and are not safe for ingestion. Eco‐friendly processes are widely researched for safe lycopene (Li, Cui, and Hu 2023; Li, Xu, et al. 2023; Li, Zhan, et al. 2023).

### 9.2 Hydrostatic Pressure Processing (HPP)

Extraction using pressure and temperature is an efficient method of extraction from tomatoes. Water and organic solvents in the slightest amounts are used in this procedure; thus, it is regarded as safe. Extraction of beta‐carotene from small carrots using HPP increases the yield (Hwang et al. 2023).

### 9.3 Supercritical Fluid Extraction (SFE)

Gases and liquids are used as solvents for extraction when subjected to high temperature and pressure, which is more than their critical value. Carbon dioxide is the most commonly used solvent. This process is safe for humans as well as the environment. It is sensitive to viscosity and fluid density. Beta‐carotene and lycopene are extracted efficiently using this technique (Bello et al. 2023).

### 9.4 Ultrasonic‐Assisted Extraction

This method produces ultrasonic vibrations similar to sound but at a high‐pitched frequency that causes the solvent to penetrate deeply into the cell, leading to adequate homogenization of solvent with pigment. The result is better quality yield in high concentrations. Lycopene extraction is sped up when this technique is used. The end product was also enhanced by 10% with ultrasonic‐assisted extraction (Yadav, Khanpit, et al. 2023; Yadav, Khare, and Dhamole 2023). Larasati, Suriani, and Nazir (2023) researched extracting beta‐carotene using this technique. They concluded that the yield of beta‐carotene was maximum when using ultrasonic‐assisted extraction.

### 9.5 Microwave‐Assisted Extraction

MAE merges electromagnetic microwaves with conventional solvent extraction. Microwaves alter the structural integrity, while traditional solvents extract carotenoids by profoundly penetrating the compound. The extraction takes place up to the maximum potential of the compound (Georgiopoulou et al. 2023). MAE is reported to use minimal time and solvents, have a greater yield rate, are relatively cheap, and produce more rapid thermal energy than all other traditional techniques. These characteristics make this technique very efficient for extracting functional biomolecules, such as the extraction of carotenoids from plants. MAE can also be used in industries (Hladnik et al. 2023).

### 9.6 Microemulsion Technique

Various surface‐acting substances known as surfactants are used in this method for the successful extraction of phytochemicals and organic molecules such as proteins, carotenoids, phenols, and enzymes from fluids, lipids, proteins, and other food products meals (İnan‐Çınkır et al. 2024). These surfactants, even in small amounts, can lower the surface tension of molecules, changing the polarity of hydrophobic substances and enhancing product yield. The emulsion of lecithin in olive oil in the presence of aqueous media is used to extract lycopene. Beta carotene is extracted by this method using short‐chain fatty acids in aqueous media (Ferrando et al. 2024).

### 9.7 Water‐Induced Hydrocolloidal Complexation

This technique combines lycopene and pectin fiber, known as complexation, by incorporating water molecules into tomato pulp. Later, the traditional solvent extraction method is used to isolate lycopene from pectin (Yu et al. 2024).

#### 9.7.1 Enzymatic Extraction

Hydrolases such as cellulase and pectinase are used to break down the components like cellulose and pectin in the cell wall. This is a safe and efficient technique for lycopene extraction. This method can be combined with other procedures, such as supercritical carbon dioxide extraction (Tran and Nguyen 2023). Beta carotene can be separated or isolated using other techniques, such as different types of chromatography. The most common methods are open column (OCC) and high‐performance liquid chromatography (HPLC). HPLC yields pure beta‐carotene, and its quantifying value is up to 97.95%. OCC is generally used to extract beta‐carotene from fruits. Owing to its non‐polarity, beta‐carotene is isolated with the help of organic solvents like hexane or chloroform (Sodedji et al. 2024). After extraction, the product is then filtered before further processing. Polar solvents like hexane are used in minimal amounts for filtration using a flask and condenser under the conditions of heat and time (Başaran, Çuvalcı, and Kaban 2023).

Pajot et al. (2023) conducted research in 2013 to study the efficacy of centrifugal partition chromatography (CPC) in extracting beta‐carotene from *Dunaliella salina*, a microalgae. The results showed its efficiency and optimization in extracting metabolites. The extraction of beta‐carotene from microorganisms is done using oils at high concentrations and pressure. Supercritical CO_2_ extraction, conventional solvent extraction with the help of organic solvents, and high‐pressure extraction are other commonly used extraction techniques for beta‐carotene (López et al. 2023). Beta‐carotene and other carotenoids can be extracted by a commonly used procedure of separation with water. Nevertheless, a hydrocarbon structure makes it highly soluble in fats, and it can dissolve in oil even at low concentrations and room temperature (Papapostolou et al. 2023). Total time, heat, and processing conditions of food used for extraction can highly impact the concentration of extracted beta‐carotene. Thermal energy provided to food during cooking procedures such as steaming, boiling, and blanching can break the linkage between beta‐carotene and proteins, thus easing its isolation (Kresnowati and Lestari 2024).

## 10 Toxicity and Side Effects

Lycopene can react within the organism, and it is dependent on the presence and reaction with other antioxidants, the location of the reaction, physiological structures, and the presence of oxygen (Wang, Heng, Song, et al. 2023; Wang, Li, Duan, et al. 2023; Wang, Lin, Liu, et al. 2023; Wang, Shen, Li, et al. 2023; Wang, Xu, Tan, et al. 2023; Wang, Zhang, Yin, et al. 2023; Wang, Zhang, Zhang, et al. 2023; Wang, Zhou, Zheng, et al. 2023). Tobacco present in cigarettes can react with lycopene, leading to the production of potentially toxic metabolites that may cause undesired side effects in the respiratory tract. The toxic effects of lycopene are dependent on the concentration of accumulating lycopene, the reaction with tobacco/alcohol, the dose administered, and the impact on cell signaling (Ibrahim and Shaheen 2023).

### 10.1 Lycopenemia

Excessive intake of tomato juice can lead to a condition known as lycopenemia, which is characterized by a significantly high intake of lycopene. This condition causes yellowish pigment in the skin, especially on the hands and feet. Lycopenemia also causes fatty deposits in the liver due to high accumulation and alters liver function (Zon, Khairudin, and Hazlan 2023). Lycopene accumulates in the epidermis's outer layer due to a fatty layer and high lycopene binding capacity. Clinical symptoms and dietary history diagnose lycopenemia. The cure is limiting lycopene‐containing food until the pigment subsides (Li, Cui, and Hu 2023; Li, Xu, et al. 2023; Li, Zhan, et al. 2023).

### 10.2 Lycopene in Pregnancy

Lycopene from food sources is safe, but supplementation and dietary intake might have adverse effects during pregnancy. Studies show that daily supplementation of lycopene up to 2 mg during mid gestation enhanced the risk of premature birth and low birth weight babies (Kang et al. 2023). Studies regarding the safety of lycopene supplements during lactation are limited. Hence, the best solution is to avoid lycopene supplementation in addition to dietary intake of lycopene‐rich sources during pregnancy and lactation. Research evaluating the toxicity of beta‐carotene in animal models exhibited that beta‐carotene does not cause cancer or mutations, produce toxic effects, or disturb fetal development in embryos. It also does not cause vitamin A toxicity (Lai et al. 2023). People ingesting high doses of beta‐carotene for health benefits are not at risk of hypervitaminosis A. Beta‐carotene has been generally regarded as safe (GRAS) by the Food and Drug Administration (FDA) to be used as a natural pigment or additive in pharmaceuticals, food products, and cosmetics (Abrego‐Guandique et al. 2023).

### 10.3 Hypercarotenemia

This condition is characterized by excessive intake of beta‐carotene, that is, more than 30 mg per day for a long duration, leading to increased blood concentrations of beta‐carotene and a pale appearance of hands and feet. This pigmentation can fade away upon restricting the intake of beta‐carotene supplements and dietary intake (Thomas, Calle, and Fernandez 2023; Thomas, Ramkumar, et al. 2023).

## 11 Digestion, Absorption, and Metabolism

**FIGURE 6:**
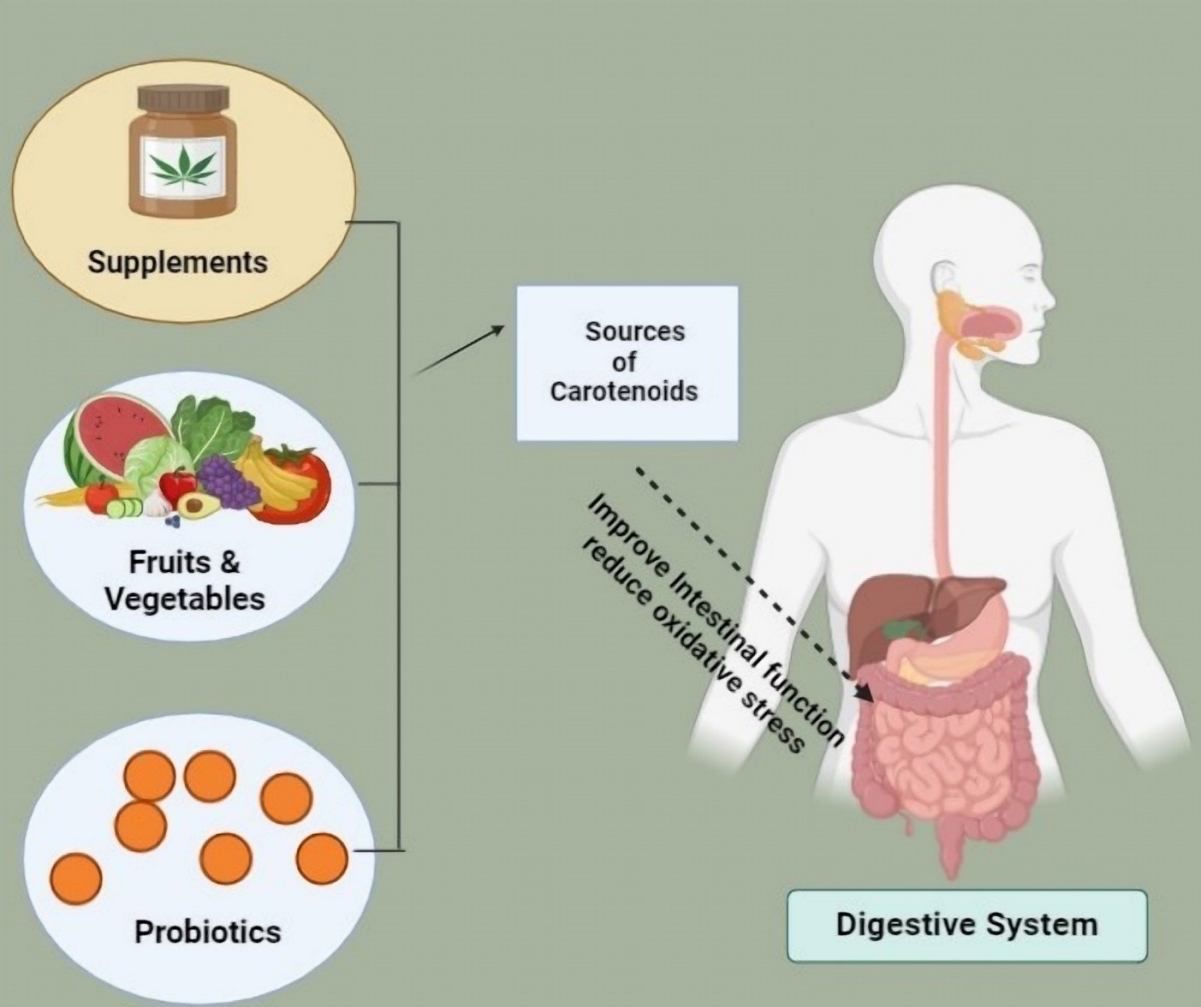
Health benefits of carotenoids for digestive system.

Only up to 30% of ingested lycopene is digested by human GIT. Lycopene digestion starts in the stomach, and the rest occurs in the small intestine with the help of enzymes released by the pancreas, such as lipase and bile salts. These enzymes degrade lycopene into absorbable form, as mentioned in Figure 6. Digestion helps to diffuse lycopene with fats (Wu, Wu, et al. 2023; Wu, Zhu, et al. 2023). This leads to the formation of small aggregates of fats with bile salts, also known as micelles. These micelles are then easily absorbed by cells of the small intestine. Cis configuration of lycopene is more bioavailable to the human gut as it shows less reluctance during digestion and absorption than trans configuration (Nunes et al. 2023). Since lycopene is liposoluble, it exhibits characteristics similar to fats and oils during digestion and absorption. At first, the lycopene is detached from the matrix, and then it forms an immiscible mixture, forming micelles that show high solubility in the intestine. These micelles easily cross the small intestine's membrane and enter the bloodstream (Wu, Wu, et al. 2023; Wu, Zhu, et al. 2023). The duodenal portion of the small intestine facilitates the formation of liposomes that are absorbed by the small intestine with the help of passive transport. The intestine membrane also contains carriers for lycopene transport that aid the absorption of the process (Alam and Kassama 2023).

**FIGURE 7:**
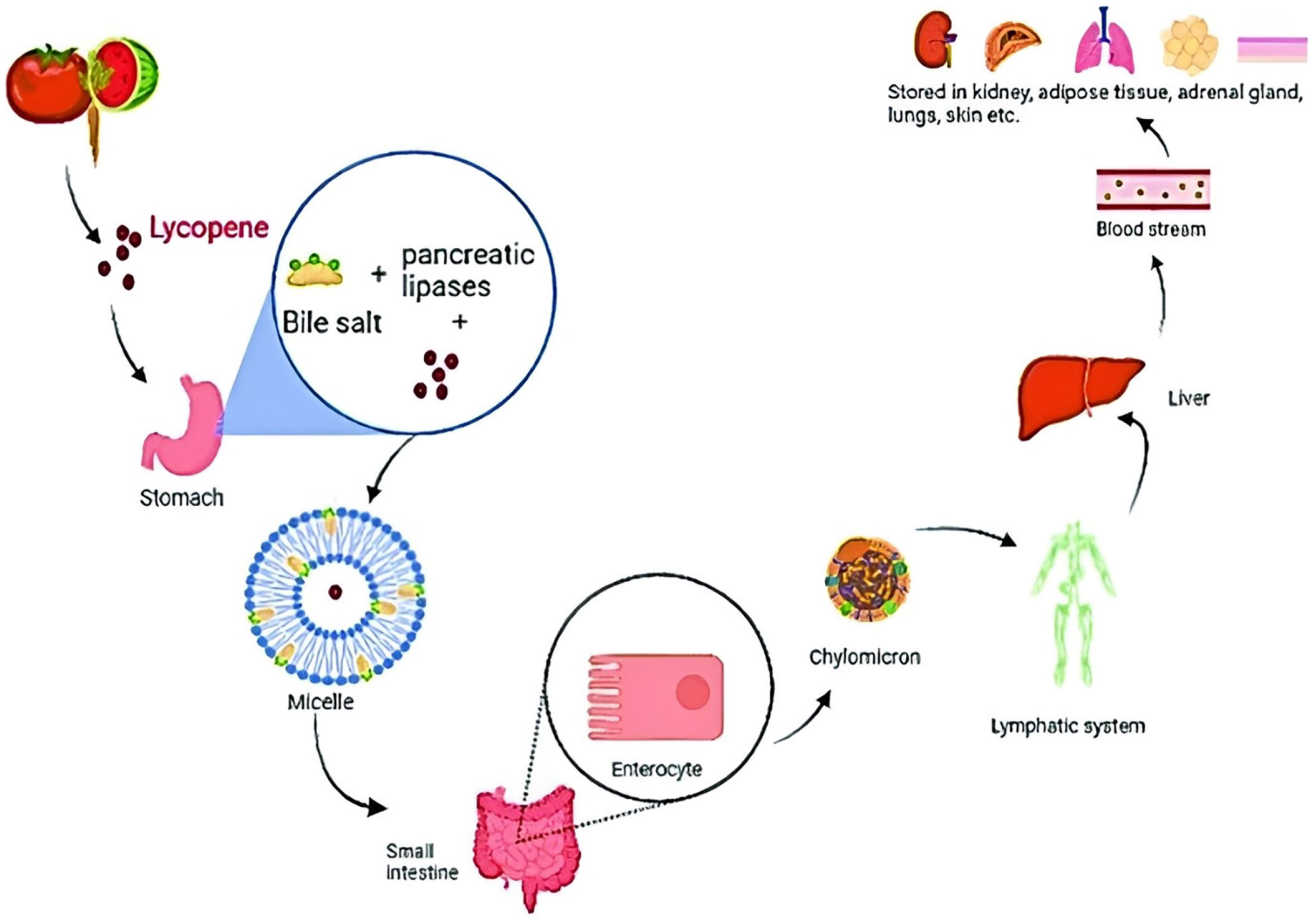
Digestion and absorption pathway of lycopene inside human body (Bin‐Jumah et al. 2022).

Digestion‐absorption pathway of lycopene (Figure 7) is completed in six phases inside the lumen. At first, lycopene detaches from the plastids of plant cells and enters the food substrate. Chewing enzymes and the presence of lipids unlink lycopene from the food matrix, making it enter the digestive tract. Lycopene mixes up with lipid droplets in acidic and enzymatic surroundings. Bile salts change the configuration of lycopene isomers to increase bioavailability. Pancreatic lipase helps release lycopene and form micelles in the intestine (Li, Cui, and Hu 2023; Li, Xu, et al. 2023; Li, Zhan, et al. 2023). Micelles comprise fats, cholesterol, and bile acids, making them liposoluble and hydrophilic. Owing to this property, lycopene, which is lipophilic, remains dissolved even in aqueous environment. Micelles are then absorbed by passive diffusion through the apical membrane of enterocytes. After absorption along with lipids, at a rate of up to 10% inside the small intestine, lycopene is divided into the surrounding tissues (Ahmad, Ibrahim, and Abdelhamid 2023; Ahmad, Khan, et al. 2023; Ahmad, Riaz, et al. 2023). Various factors, such as medicines, fibers, other food components, and phytosterols in GIT, influence lycopene absorption. Lycopene is reported to bind with them, and thus, its absorption is hindered. Some studies show that absorption may reduce up to 40% (Li, Cui, and Hu 2023; Li, Xu, et al. 2023; Li, Zhan, et al. 2023). Epithelial cells of the small intestine take these micelles with the help of diffusion or membrane proteins. Micelles are converted to chylomicrons, which are broken down and are encapsulated. These are transported to blood circulation and lymphatic vessels, which diffuse these throughout the blood and body (González‐Peña et al. 2023).

Lymphatic vessels carry lycopene to the liver after absorption from the gut. LDL and VLDL attach with lycopene and make insoluble complex compounds. These complexes then enter the bloodstream and are carried to various organs. In fasting conditions, LDL and VLDL transport 76% and 7% of lycopene in the liver, respectively, while HDL is responsible for 17% of that transport (Guo, Liu, and Luo 2024). The liver is the prime storage organ of lycopene, which also metabolizes it under low lycopene concentrations in blood. Organs with high amounts of fat, such as adipose tissues, reproductive tissues, adrenal glands, and pancreas, also contain lycopene in high concentrations (Abdel‐Naim et al. 2023). Lycopene is mainly excreted through the body via the fecal route, and small concentrations are excreted via sweat and the urinary tract. Breast milk also contains a significant amount of lycopene, that is, up to 3.8 mg per 100 g which is 10% of serum content (Reboul 2023). When consumed through food, human blood contains up to 1 μmol per liter of lycopene. The usual average is 0.6 μmol per liter, up to 6% of total plasma. The serum concentration of lycopene decreases with age, and older adults have lower lycopene levels than adolescents having close dietary patterns and ethnicity (Abir et al. 2023). Advanced age brings various acute and chronic conditions related to GIT health, such as the problem with stomach acid levels, diseases of the gut, and lumen enzyme concentration that may lead to hindrance in the normal absorption of carotenoids (Wang, Heng, Song, et al. 2023; Wang, Li, Duan, et al. 2023; Wang, Lin, Liu, et al. 2023; Wang, Shen, Li, et al. 2023; Wang, Xu, Tan, et al. 2023; Wang, Zhang, Yin, et al. 2023; Wang, Zhang, Zhang, et al. 2023; Wang, Zhou, Zheng, et al. 2023). Naturally, beta‐carotene is present primarily in all trans configurations in plant sources. Thermal processing of food usually induces conversion into cis‐form. 9‐cis, 13‐cis, and 15‐cis are mostly found in isomeric forms. The chewing process can detach beta‐carotene from the food matrix and increase its isolation by up to 35%. This has been proven by various studies conducted on beta‐carotene digestion (Muteeb et al. 2023).

Low gastric pH in the acidic range can cause beta‐carotene loss to a small extent. A complex of beta‐carotene with cations is formed, which may further cause the production of trans‐cis mixed isomers. Stomach lipase helps to digest up to 40% of consumed fats, and it also helps to disintegrate triacylglycerols found in beta‐carotene as well as fats (Wang, Heng, Song, et al. 2023; Wang, Li, Duan, et al. 2023; Wang, Lin, Liu, et al. 2023; Wang, Shen, Li, et al. 2023; Wang, Xu, Tan, et al. 2023; Wang, Zhang, Yin, et al. 2023; Wang, Zhang, Zhang, et al. 2023; Wang, Zhou, Zheng, et al. 2023). After isolation from the plant matrix, beta‐carotene forms bile salt micelles similar to lycopene and is later taken up by the intestinal membrane. Lipids in food and bile are important for producing micelles containing beta‐carotene (Liu, Lin, et al. 2023; Liu, Zhou, et al. 2023). These micelles are converted to chylomicrons inside the lumen, transporting them inside vessels. The liver is the primary storage organ for beta‐carotene as it is also responsible for the metabolism and storage of vitamin A. Undesired beta‐carotene is excreted via bile and further via the fecal route after metabolism (Satyanarayana 2024). Up to 5 g of fat is reported to be adequate for beta‐carotene absorption. Food processing, formulation, enzyme release, presence of food in the gut, and bile interfere with the absorption process, and thus, the bioavailability of beta‐carotene cannot be accurately conferred on account of consumption from various plant sources (Liu, Lin, et al. 2023; Liu, Zhou, et al. 2023). The maximum absorption of beta‐carotene by the gut is reported to be up to 65%. Its absorption can be evaluated by dispensing specific concentrations of beta‐carotene to the subject and then measuring beta‐carotene content in feces later. Absorbed beta‐carotene will equal the subtraction of fecal content from the dose given (Zhou et al. 2024). Processing of food enhances beta‐carotene absorption, probably due to changes in isomerization. Studies revealed that the concentration and the kind of ingested fiber reduce the absorption of beta‐carotene. Although cis configuration is reported to make more micelles than trans configuration of beta‐carotene, cells tend to take trans isomers up more than cis ones (Chen, Capuano, and Stieger 2024).

## 12 Health Benefits

Owing to the provitamin A activity of dietary carotenoids, they have gained enormous fame for their functional properties and wide‐ranging health benefits. Lycopene does not possess a beta‐ionic ring in its structure; thus, it does not exhibit provitamin A activity. The functional importance of lycopene is due to its efficient oxygen‐scavenging role among all other carotenoids (Sombié et al. 2023). Beta‐carotene is the precursor and primary source of vitamin A. Adequate consumption of beta‐carotene leads to antioxidant activity and various associated biological benefits (Ansah et al. 2023). The health effects of lycopene and beta‐carotene have been summarized in Table 3 and explained in the upcoming literature. Some major health‐promoting benefits are associated with lycopene and beta‐carotene and their mode of action in various diseases.

**TABLE 3:** Studies related to biological effects of lycopene and beta‐carotene.

Condition/Disease	Lycopene effect	Beta‐carotene effect	Reference
Oxidative damage	Prevents oxidation of DNA and genetic material, activates antioxidant response element, and enhances antioxidant phase 2 cytoprotective enzymes	Prevents lipid peroxidation of cellular membranes and adds superoxide radicals	Cao et al. (2021), Althurwi et al. (2022)
Cancer	Prevents oxidative damage to DNA, interrupts cell cycle in cancerous cells, downregulation of apoptosis, and slows down cancer progression	Regulates NF‐kB, inhibits cell growth, and promotes apoptosis	Saini et al. (2020), Kordiak et al. (2022)
Oral diseases (periodontitis)	Suppresses saliva and reduces probing depths	Reduces probing depths, targets NF‐kB, and shows anti‐inflammatory properties	Sanadi et al. (2024), Nilesh et al. (2021)
Cardiovascular diseases	Reduces lipid peroxidation, reduces LDL and TGL levels, inhibits cholesterol synthesis in macrophages, and reduces production of AGE	Reduces CRP levels, ROS, nitrotyrosine and downregulates NF‐kB	Przybylska and Tokarczyk (2022), Yang et al. (2022)
Inflammatory diseases	Regulates signaling pathways, inhibits TNF‐α, and stimulates IL‐10	Inhibits NF‐kB activation, IL‐8, and cyclooxygenase enzymes responsible for inflammation	Saini et al. (2020), Cheng et al. (2021)
Fat reduction	Changes HDL 2 and 3, anti‐inflammatory reactions and increases adiponectin	Slows maturation of adipocytes and controls adiposity	Albrahim and Alonazi (2021), Marcelino et al. (2020)
Male infertility	Increases sperm motility and prevents oxidative damage	Balances ratio of omega 3 and omega 6	Antonuccio et al. (2020), Dutta et al. (2022)
Skin diseases	Prevents damage by UV rays, prevents inflammation, and alters signaling pathways	Reduces lipid peroxidation and modulates cell signaling	Zhang et al. (2022), Maretti et al. (2021)
Immune system disorders	Prevents T cell inactivation and reduces proinflammatory cytokines	Reduces CRP levels and modulates cytokine production	Imran et al. (2020), Chen et al. (2021)
Diabetes mellitus	Prevents oxidative damage to pancreas, improves glucose metabolism, and prevents complications	Improves insulin resistance, glucose metabolism, and hyperglycemia	Leh and Lee (2022) Marcelino et al. (2020)
Neurodegenerative diseases	Prevents lipid oxidation in neural membranes, misfolded protein accumulation, and inhibits cognitive and mitochondrial dysfunction	Reduces beta‐amyloid plaques, binds with acetylcholine esterase, and improves cognitive impairment	Saini et al. (2020), Kabir et al. (2022)
Skeletal diseases	Reduces free radical damage to bone tissues, prevents osteoporosis, inhibits osteoclasts, and improves osteoblasts	Reduces pit formation and osteoclast synthesis, and increases bone mass	Walallawita et al. (2020), Gao and Zhao (2023)
Liver diseases	Inhibits oxidative damage to liver, reduces aflatoxin‐related injury	Reduces aflatoxin toxicity and hepatic fat accumulation, and modulates NF‐kB	Ibrahim et al. (2022) Clugston (2020)
Eye diseases	Lowers plaque buildup and oxidative damage to retina	Scavenges free radicals and acts as anticataract agent	Johra et al. (2020), Choo et al. (2022)

### 12.1 Antioxidant Potential

Volatile, reactive, and transitory molecules with a free valent electron are called free radicals that can damage anything they come in contact with. This damage can further lead to various adversities such as mutations, cardiovascular problems, and other disorders. Oxygen‐based free radicals are the most common (Lin et al. 2023). Stress is the body's natural response to any unusual disturbance. It might lead to an imbalance in the physiological system due to the interaction of negative stimuli with defense mechanisms. Oxidative stress is caused by free radicals when the body's defense mechanism cannot counter control them, thus leading to an imbalance. Oxidative stress is known to impair normal functions of the body and cause various diseases due to free radical damage, cellular injury, and death (Chaudhary et al. 2023). Antioxidants are the compounds that counteract free radicals. Lycopene is one of the few naturally occurring liposoluble antioxidants. It can halt lipid oxidation as it is a strong scavenger of singlet oxygen (Caiati, Stanca, and Lepera 2023).

Lycopene is reported to possess the greatest antioxidant potential among all carotenoids, such as beta‐carotene, lutein, beta‐cryptoxanthin, etc. Lycopene has 2× and 100× more antioxidant potential than beta‐carotene and vitamin E (López‐Valverde et al. 2024). When beta‐carotene and lycopene were studied in albumin and dipalmitoylphosphatidylcholine (DPPC), they exhibited free radical quenching ability of the same nature. Both beta‐carotene and lycopene were reported to cause oxidative degradation of fats, primarily unsaturated fatty acids in cell membranes and LDL, as they are highly liposoluble (Saejung, Lomthaisong, and Kotthale 2023). These carotenoids can inactivate various free radicals by using three main mechanisms. These mechanisms include the transfer of electrons, hydrogen atoms, and adduct formation. The latter two are responsible for the free radical quenching activity of lycopene. Carotene complexes inside the membrane lack polarity, but the polarity of the surrounding matrix helps in adduct formation and transfer of hydrogen atoms. The reaction of lycopene with ROS is impacted by the kind of ROS, configuration, and location of lycopene inside the cell membrane (Wang, Heng, Song, et al. 2023; Wang, Li, Duan, et al. 2023; Wang, Lin, Liu, et al. 2023; Wang, Shen, Li, et al. 2023; Wang, Xu, Tan, et al. 2023; Wang, Zhang, Yin, et al. 2023; Wang, Zhang, Zhang, et al. 2023; Wang, Zhou, Zheng, et al. 2023). The interaction between free radicals and carotenes varies according to structural characteristics, the isomerization of carotene, and the type of free radical. Lycopene is known to deactivate singlet oxygen through physical interactions with it. Chemical interactions only account for up to 0.05% of lycopene's total antioxidant activity (Yildiz et al. 2023).

Lycopene and ROS can interact in multiple manners. Lycopene can formulate and isolate other antioxidants with the help of radicals, such as vitamin C from semidehydroascorbyl radicals. Hence, lycopene protects the cells by producing free‐radical quenching compounds (Islam et al. 2024). Lycopene also protects other antioxidants against oxidation. The antioxidant potential of lycopene is attributed to oxidation–reduction reactions. A low concentration of lycopene exhibits antioxidant activity, while a high concentration leads to the induction of more oxidative stress. The partial pressure of oxygen, interaction with other compounds, and lycopene load in the tissue can affect pro‐oxidant activity (Kulawik, Cielecka‐Piontek, and Zalewski 2023). Lycopene's pro‐oxidance can benefit already injured tissues, thus reducing the chances of mutations and their development and preventing further damage to these cells (Georgaki et al. 2023). The primary role of lycopene as an antioxidant is to prevent oxidation of genes and DNA so that the hereditary material is safe from mutations and does not cause disorders in the body. The linear structure of lycopene with multiple double bonds is responsible for its effective antioxidative potential (Macar et al. 2023).

Lycopene is reported to regulate the enzymes that catalyze conjugation reactions during detoxification processes. These enzymes regulate genetic activity, immunity, and cell cycle. This leads to activation of response to oxidation, which helps synthesize enzymes important for detoxification and cell protection (Macar et al. 2023). Beta‐carotene's antioxidant potential relies on adding superoxide ions, while lycopene reacts with these ions by transferring electrons. Churchill et al. (2023) reported that lycopene and beta‐carotene show less superoxide free radical quenching power when compared to xanthophylls. Beta‐carotene, like lycopene, prevents fat oxidation inside cell membranes by producing a stable radical. This activity has also been seen in other antioxidant carotenoids. Beta‐carotene is also known to inactivate CCl4 radicals. Beta‐carotene protects the lipid bilayer's vesicles by inhibiting free radical formation and preventing oxidation inside the membrane. Peroxyl radicals are found to be degraded when adequate oxygen pressure is present inside the membrane, along with small amounts of beta‐carotene (Nazar et al. 2023).

### 12.2 Cancer

The production of abnormal cells due to unchecked cell division and multiplication leads to cancer. Free radical damage has been reported to play a key role in the development of chronic diseases such as cancer, metabolic diseases, and cardiovascular disorders (Sadiq 2023). Numerous research studies have proven the positive impact of functional food and bioactive ingredients in gene regulation, cancer prevention, and treatment. Lycopene is one such molecule that interferes with cancer advancement by halting genetic mutations and the spread of cancer (Ozkan et al. 2023). The bioavailability of lycopene is important for its role as a free radical quencher and for preventing damage to vital components such as genetic material. Lycopene also aids in controlling intracellular communication and expression of genes that may lead to mutations (Minhas et al. 2024). The antitumor activity of lycopene may be due to its ability to boost immunity; however, limited literature is present to prove this. Research conducted on women concluded that lycopene from tomatoes inhibited free radical stress in the DNA of white blood cells to a great extent. Hence, lycopene shields genetic material, body structures, and compounds against ROS damage. It also plays a prime role in regulating the production of chemical messengers, programmed cell death, cell division, and metabolic processes of carcinogenic compounds (Boulaajine and Hajjaj 2024).

Lycopene is reported to aid in synthesizing stage 1 and 2 enzymes required during the metabolic degradation of cancer‐causing agents in the body. Enzymes produced in stage 1 trigger the activation of these agents, and enzymes released in stage 2 aid the excretion of these agents by bonding them to excreting agents. Lycopene also hinders the production of cancerous cells, mutations, and toxicity‐related damage in the body (Bradley et al. 2023). Somatomedin C or IGF are related to cancer progression. Lycopene inhibits it, thus reducing the risk of cancer and acting as an effective free radical scavenger. Various animal studies and cellular culture research have pointed out that the concentration of lycopene in plasma is inversely proportional to the likelihood of carcinogenesis (Boguszewski, da Silva Boguszewski, and de Herder 2023). Lycopene therapy is effective against tumors of the respiratory tract and the upper part of the digestive tract. Along with other antioxidants, such as phytochemicals and vitamins, lycopene is reported to protect from these cancers. Colon and rectal tumors are the most common cancers of the digestive tract. Omega‐3 fatty acids, in conjunction with lycopene therapy, hinder uncontrolled cell division and multiplication when given at appropriate high doses (Saejung, Lomthaisong, and Kotthale 2023). Serum carotenoid levels are reduced in people with colon polyps. In a research study, lycopene was reported to be reduced by 35%. In contrast, compared to normal individuals, beta‐carotene was reduced by a percentage in these adenomatous patients. Hence, incorporating these carotenoids at the beginning can reduce the development of tumors (Lüdtke et al. 2023).

Among all cancers, lycopene is more efficient against prostate cancer. A study conducted by Harvard students proved that people ingesting sufficient amounts of lycopene had a lower likelihood of developing tumors such as prostate tumors (Kusdemir et al. 2023). Dhillon, Deo, and Fenech (2023) studied the inverse association between lycopene ingestion and cancer risks. They concluded that subjects consuming lycopene in appreciable amounts through dietary sources had 44% fewer chances of getting tumors and 25% fewer chances of developing prostate cancer specifically. Gupta et al. (2024) reported in their research that treatment with lycopene had a beneficial effect in preventing prostate cancers that do not respond to hormonal therapy. Lycopene is a safer treatment than other modes of cancer therapies. Thus, it should be started in the early phases of cancer. Rahman et al. (2023) studied the effects of lycopene intake on IGF and saw a notable reduction in the development of cells that came in contact with lycopene. Cancers of female reproductive organs and breasts rely on hormones and show a response to lycopene therapy.

Another study by Chalabi et al. focused on genes involved in breast carcinogenesis and their mutation. They concluded that lycopene from plants directly impacts gene mutations, thus establishing this carotenoid's gene regulatory effect. Lycopene can get attached to cancerous cells and halt the further progression of tumors. Sufficient consumption of lycopene from watermelon reduces the likelihood of cervical cancer in women by fivefold. Lycopene can also control cell signaling and hormonal modulation that assist in the progression of tumors and their severity, such as that of the uterus (Shahiwala and Khan 2023). In a randomized controlled trial, subjects were given plant juice containing significant lycopene for a fortnight and later were exposed to ozone. Lycopene quantity in lungs increased by 12% while DNA present in alveolar epithelium reduced by 20% in the controlled group compared to the placebo group (de Carvalho et al. 2023). Ozkan et al. (2023) conducted a study on ferrets that were given varying concentrations of dietary lycopene after being subjected to cigarette carcinogens. Lycopene at all concentrations halted the downregulation of programmed cell death and cell differentiation in epithelium caused by cigarettes. Hence, lycopene can prevent lung cancer by enhancing timely programmed cell death and inhibiting uncontrolled cell division and multiplication. Numerous studies were conducted at the end of the 20th century that linked beta‐carotene consumption with reduced risk of epithelial cancers, especially lung carcinomas.

Letafati et al. (2023) are reported to authenticate a positive correlation between beta‐carotene and reduced carcinogenesis via their studies. However, some studies proved otherwise. A beta‐carotene trial with α‐tocopherol revealed that beta‐carotene also possesses carcinogenic capabilities when taken with vitamin A or E. It can increase lung carcinomas and death rates in patients routinely exposed to smoke and occupational toxins (Mourtala et al. 2023). Meta‐analysis of Yang et al. (2023) concluded that beta‐carotene in supplemental form does not prevent or protect from risks of major cancers such as prostate, lung, breast, etc. Beta‐carotene in small concentrations was reported to reduce the likelihood of carcinogenesis in groups that were women dominant.

According to Sable and Shields (2023), a beta‐carotene supplement can decrease the possibility of prostate cancer in males with low concentrations of beta‐carotene in their blood. Beta‐carotene can protect from lung carcinomas when the supplementation is started before cancer development or in the early stages of the process. Carotenoids like beta‐carotene exhibit anticarcinogenic potential in breasts due to their ability to stimulate enzyme production for detoxification, immune‐regulating and boosting potential, halting uncontrolled cell division, reducing oxidative damage to tissues, and regulating cell signaling pathways. The antioxidant potential of beta‐carotene makes it useful in helping stop the cell proliferation and formation of new vessels, regulating programmed cell death and immunity. Beta‐carotene exhibits anticarcinogenic function by controlling redox reactions of sequence‐specific DNA‐binding factors such as NF kb (Didier et al. 2023).

### 12.3 Oral Diseases

Carotenoids are also reported to play a significant role in diseases of the mouth, such as:

#### 12.3.1 Periodontitis

This is a severe infection of gums and other tissues surrounding teeth. A gram‐negative bacteria, *Porphyromonas gingivalis* is responsible for causing this disease and leading to progressive breakdown of bones surrounding teeth sockets (Radzki et al. 2024). Lycopene intake decreases interleukin concentration in saliva, thus reducing the risk of oral infections. The ingestion of lycopene is promising for managing periodontitis. In all patients with this infection, lycopene caused a notable decrease in probing depth (Boloor et al. 2023). Beta‐carotene also showed beneficial effects against periodontitis by reducing the synthesis of immune cytokines produced in white blood cells, thus showing functionality by regulating transcription factors like NF kb (López‐Valverde et al. 2024). Moreover, beta‐carotene from plant intake causes a reduction in probing depths after therapy in non‐smoker patients with severe infections. Low plasma concentrations of beta‐carotene were reported in periodontal patients having chronic infection. Beta‐carotene in adequate concentrations in the plasma is reported to protect against infections and inflammatory cascades. Thus, its potential disease‐preventive role should be considered while planning dietary intake for these patients (Li, Yue, and Xiao 2024).

#### 12.3.2 Leukoplakia

The formation of white patches inside the mouth is known as leukoplakia. It is related to oral dysplasia, which might lead to cancer formation. Tobacco intake for long‐term durations is known to cause leukoplakia. Nitrous reactive species present in tobacco cause cancer. In these individuals, antioxidant therapy can help reduce the risk of cancer and halt the conversion of pre‐cancer cells into cancerous cells (Tovaru et al. 2023). Lycopene is reported to possess the most singlet oxygen antioxidant potential among carotenoids. Thus, it can effectively treat and prevent oral leukoplakia without causing any harm. Lycopene regulates genes involved in cell signaling other than free radical scavenging property. Synthetically produced beta‐carotene administered at high concentrations seems beneficial in treating leukoplakia (Ahmad, Ibrahim, and Abdelhamid 2023; Ahmad, Khan, et al. 2023; Ahmad, Riaz, et al. 2023).

#### 12.3.3 Lichen Planus (LP)

This is an incurable inflammatory condition of mucous membranes inside the mouth. It only affects up to 4% of people and usually occurs in the inner linings of the skin or mouth. Patients suffering from LP, where the cells started to break down and shrink, had deficient plasma concentrations of lycopene (Motahari, Daliraan, and Poorzare 2023). Reduced consumptions of antioxidants are linked with the development of this disease, but free radical damage seems to be a significant parameter in disease progression. Owing to the antioxidant ability of lycopene, it can aid in protection from LP. Beta‐carotene also shows healing properties for LP lesions by reducing oxidative stress. It is effective in severe LP with significant cell breakdown to some extent (Gupta, Nath, and Mangalathillam 2023).

#### 12.3.4 Oral Submucous Fibrosis (OSMF)

OSMF is a precancerous disorder of the mouth that causes inflammation, leading to the thickening and scarring of oral tissues. It is mostly common in the Asian subcontinent. Studies have shown that lycopene can hinder fibrosis in liver cells. It might play the same role in halting oral fibrosis. Lycopene is also known to enhance protection against stress‐related damage and hinder inflammation (Nigam et al. 2023). Pérez‐Leal et al. (2024) pointed out that lycopene and other carotene aid in improving the ability to open the mouth in OSMF patients. Blood levels of beta‐carotene were reported to be very low in these individuals with OSMF. The concentration decreased further with the advancing stages. Beta‐carotene supplementation for 1.5 months increased blood concentration of beta‐carotene and reduced serum malondialdehyde levels that indicate oxidative stress.

#### 12.3.5 Cardiovascular Diseases (CVDs)

Cardiovascular disorders have been ranked as the topmost diseases responsible for the highest morbidity and mortality. Hypertension, vascular remodeling, hypercholesterolemia, atherosclerosis, and cigarette smoke are a few etiological factors in the development and severity of CVDs. A plant‐based diet low in fats and rich in fruit and vegetable phytochemicals is reported to reduce the likelihood of CVD. Low serum levels of lycopene are linked with the development of high blood pressure, heart attack, and stroke. Serum lycopene levels are associated with a lower risk of heart‐related conditions (Kulawik, Cielecka‐Piontek, and Zalewski 2023). Free radical damage plays a primary part in instigating cardiovascular diseases, their progression, and their severity. Lycopene therapy in CVD patients targets biomarkers of oxidative stress and inhibits lipid peroxidation, thus enhancing cellular oxygen supply and blood flow by the end of the regime. Consumption of antioxidant supplements such as carotenoids significantly affected disease prognosis and overall cardiac health (Okoi et al. 2024). Lycopene's antioxidant potential exerts a vital impact on lipid peroxidation. A study was conducted on normal individuals who consumed lycopene from various sources, and one group had a placebo. Lycopene‐consuming individuals exhibited a reduction in low‐density lipoprotein oxidation. Moreover, serum TGL and LDL were also reduced in these individuals, along with the reduction in lipid peroxidation due to lycopene, immunoglobin G, an important biological parameter of CVD‐related free radical damage and inflammation, seemed to reduce by three times (Jurado‐Fasoli, Mesas‐Fernández, and Rodríguez‐García 2023).

Various studies support lycopene protection against CVD. Low serum concentrations of lycopene are reported to be associated with poor CVD‐related prognosis and high death rate. Supplements can increase serum lycopene concentration, decrease oxidative damage, and enhance antioxidant concentration (Zhang, Chen, et al. 2023; Zhang, Zhai, et al. 2023). Guo, Huang, and Li (2023) study revealed a new pathway through which lycopene exerts its protective effects against CVD. Lycopene can regulate cholesterol inside the body, possibly due to having the same course of action in the early stages. Lycopene is also reported to halt cholesterol production in white blood cells by downregulating β‐hydroxy β‐methylglutaryl‐CoA reductase enzyme and its genetic expression. Macrophages prefer lycopene over other carotenoids to halt cholesterol synthesis due to its better potential. Cellular uptake of lipoproteins is seen more in white blood cells containing antioxidants such as carotenoids than in high amounts of cholesterol. Lycopene and beta‐carotene reduce cholesterol production in the body by increasing LDL excretion from blood after activating membrane receptors of LDL. Lycopene aids in decreasing the production of advanced glycated end products that consequently help in cardiac protection. Lycopene also helps decrease oxidative damage to cellular structures and genetic material, inducing vasodilation, enhancing membrane functioning, and enhancing cell access to nitric oxide. Consumption of up to 5 medium tomatoes or supplements containing up to 15 mg of lycopene every day was reported to impact hypertension and reduce blood pressure to a great extent (Yadav, Khanpit, et al. 2023; Yadav, Khare, and Dhamole 2023). Increasing the consumption of carotenoids like lycopene and beta‐carotene reduces the likelihood of coronary heart disease (CHD). Research shows the effect of beta‐carotene in reducing the prognosis of CVD, particularly myocardial infarction (Sanadi et al. 2024). Various studies showed low blood levels of beta‐carotene in patients with high c‐reactive protein in their system, which predicts CVD. Beta‐carotene supplementations reduce free radical production and nitrotyrosine levels in the blood, hence increasing nitric oxide concentrations, which protects against the prognosis of CVD. On the other hand, it also decreases the transcription of the NF kb gene and the interaction of endothelial cells with leukocytes (Mirahmadi, Aghasizadeh, Esmaily, et al. 2023; Mirahmadi, Aghasizadeh, Nazifkar, et al. 2023).

#### 12.3.6 Inflammatory Diseases

Lycopene reduces inflammatory response and oxidative stress by two mechanisms. It either modulates cellular signaling, which induces various inflammatory mediators' responses, or regulates gene expression responsible for maintaining oxidative balance in the body (Fang et al. 2024). Conditions like diabetes mellitus (type 2) and insulin resistance are considered inflammatory conditions as proinflammatory cytokines increase, causing impairment in the body. These inflammatory cytokines are interleukins, C‐reactive proteins, and tumor necrosis factor‐alpha (TNF‐α) (Li, Cui, and Hu 2023; Li, Xu, et al. 2023; Li, Zhan, et al. 2023). Lycopene downregulates proinflammatory cytokines such as TNF‐α while inducing the production of anti‐inflammatory cytokines such as interleukin‐10 (IL‐10). Various signaling molecules and proteins are involved in the inflammatory process. Anti‐inflammatory activities of lycopene are attributed to its ability to regulate the pathway of cyclooxygenase and lipoxygenase enzymes, synthesis, and regulation of cellular signaling molecules such as nitric oxide synthase and intervene in the activities of transcription factor proteins such as NF‐kB and activator protein‐1 (Ba et al. 2023). Research on mice exhibited that lycopene prevents brain damage due to inflammatory reactions by downregulating the main factors responsible for inflammation, that is, IL‐6 and TNF‐α.

Another research conducted on mice ingesting high levels of fats revealed that lycopene reduces the inflammatory response in the body by downregulating the actions of key inflammatory mediators, importantly CRP, TNF‐α, and IL‐1β. Thus, lycopene helps manage fat build‐up and inflammatory disorders (Wang, Heng, Song, et al. 2023; Wang, Li, Duan, et al. 2023; Wang, Lin, Liu, et al. 2023; Wang, Shen, Li, et al. 2023; Wang, Xu, Tan, et al. 2023; Wang, Zhang, Yin, et al. 2023; Wang, Zhang, Zhang, et al. 2023; Wang, Zhou, Zheng, et al. 2023). Beta‐carotene is another important carotenoid that plays a part in managing inflammation. It inactivates the transcription factor protein NF‐kB, which is important for IL‐8 activity and regulation of its expression in stomach epithelium. IL‐8 initiates an inflammatory response by signaling immune cells to reach the damaged tissue (Ebadi et al. 2023).

Beta‐carotene also hinders the signaling of NF‐kB and MAPK in this tissue, thus downregulating the production of cyclooxygenase 2 and nitric oxide synthase, which were originally responsible for causing inflammation (El‐Baz et al. 2023). Nitric oxide synthesized by the enzyme nitric oxide synthase leads to tissue damage in the stomach as it produces peroxynitrite, an unstable potent oxidant responsible for various inflammatory pathways. Hence, beta‐carotene prevents oxidative damage and inflammation (Hsu et al. 2024).

#### 12.3.7 Body Fat Reduction

Carotenoids have been reported to play a role in fat reduction. They directly affect fat‐storing tissues of the body, known as adipose tissues, thus managing fat accumulation and storage.

Lycopene can modulate high density lipoproteins 2 and 3 upon routine ingestion in overweight people (Eroglu et al. 2023). A study on women given lycopene from a tomato juice source every day for 2 months concluded that lycopene intake considerably enhanced serum lycopene levels, serum triglycerides, and adiponectin (Li, Cui, and Hu 2023; Li, Xu, et al. 2023; Li, Zhan, et al. 2023). On the other hand, lycopene was seen to reduce blood cholesterol, adipokines, and waist circumference, thus causing a decrease in overall weight and body mass index (Del Castillo Vidal et al. 2023). Animal‐based research demonstrated that owing to the free radical scavenging and anti‐inflammatory potential of lycopene, it can decrease the accumulation of fats in the liver, thus reducing fatty liver disease via the activity of sirtuin 1 and regulation of gene expression (Long et al. 2024).

Beta‐carotene plays a part in fat modulation by showing activity in adipocytes. It helps maintain oxidative balance, upregulate the production of anti‐inflammatory mediators, and manage lipid metabolism in these cells. Beta‐carotene also hinders and decreases the maturation of adipocytes. It can exert its effect in various stages of the cell cycle of adipocytes and inhibit cell differentiation (Letafati et al. 2023). All of the above literature suggests that carotenoids can affect fat accumulation and metabolism in the body. Various studies concluded that ingestion of carotenoids in appreciable amounts or high serum concentration relates to low body fat (Madore et al. 2023).

#### 12.3.8 Male Infertility

Infertility is a rising issue in the modern world, and more than half of cases are due to male factors. Free radicals can damage sperm‐producing cells, thus leading to infertility. Studies report that blood concentrations of carotenoids, lycopene, beta‐carotene, and vitamin A are reduced in sterile men, and their DNA strands are more prone to breakage (Winarni et al. 2023). Freni et al. (2023) conducted a study to determine the effect of free radicals, the presence of antioxidants, and iron supplementation on fertility. This study concluded that lycopene positively affects fertility as it enhances sperm motility, oxidative balance, and the activity of mitochondria. Infertility is reported to be linked to omega‐3 ratio with omega‐6 imbalance in the body. Daily lycopene supplementation for up to 12 weeks can increase the ratio of omega3/omega‐6 (Chauhan et al. 2023).

#### 12.3.9 Skin Diseases

Skin diseases are usually a result of oxidative damage or inflammation. The skin has various defense mechanisms that can be improved by lycopene as it leads to the production of prostaglandin, which deals with tissue injury, and phospholipids that protect the cell. Thus, lycopene can decrease inflammation (Honda 2023). A study showed that fluid buildup and redness of mice ear‐skin were noticeably reduced when lycopene was administered. It was more effective than steroidal treatment. Ultraviolet rays are reported to cause skin damage, which may lead to cancer. Lycopene helps avoid this damage by downregulating epidermal ornithine decarboxylase enzyme that can lead to tumorigenesis (Lopes and Reed 2009). It also decreases tissue inflammation, thus maintaining normal cell division by protecting DNA from mutations (Crupi et al. 2023).

Lycopene and beta‐carotene both exert positive health effects related to the skin. They can improve atopic dermatitis manifestations by increasing anti‐inflammatory cells in the upper layer and enhancing the skin's appearance, hydration, and thickness. The skin contains various free radical scavengers, but free radicals are produced so excessively that the skin can no longer prevent oxidative damage efficiently (Biskanaki et al. 2023). Consumption of antioxidant supplements like vitamin C, lycopene, etc., can smoothen the skin, halting the aging of the skin and the appearance of fine lines. As sunblock helps prevent photodamage to the skin by topical application, dietary lycopene can also prevent damage due to ultraviolet radiation (Li, Cui, and Hu 2023; Li, Xu, et al. 2023; Li, Zhan, et al. 2023). Beta‐carotene is reported to be efficient in preventing photo‐sensitive skin damage and disorders. It can also prevent skin cancer by reducing oxidative degradation of fats present in the skin or inhibiting lipoxygenase enzymes, thus reducing inflammation (Bashir et al. 2024).

### 12.4 Immunoregulation

The immunoregulatory activities of carotenoids are mainly due to antioxidant potential. Lycopene is reported to prevent free radical damage of phagocytes, thus enhancing specific and non‐specific immune responses (Inoue et al. 2023). A research study on mice with compromised immune systems revealed that lycopene consumption regulated peripheral CD4+/CD8+ ratio near to normal, and T lymphocyte levels were also normalized. This may explain the immunoregulatory effect of lycopene (Zhang et al. 2024). Lycopene is a more potent antioxidant than beta‐carotene, and it can prevent free radical damage and programmed cell death of macrophages. Lycopene also suppresses tumor‐causing genes such as P53 and P21. A study on rats revealed another immunoregulatory mechanism: rats were fed lycopene supplements for 2 months, and the results concluded that levels of pro‐inflammatory cytokines and immunoglobins were reduced in prostate cells. Lycopene can also induce differentiation of white blood cells by immunomodulating the activation of lymphocytes (Mohammadzadeh, Kiani, and Amiri 2023).

Beta‐carotene can increase immunity in humans. High serum carotenoid levels correlate with slowed changes in immune functions upon ingesting beta‐carotene from vegetable juice. Studies reveal that carotene from food sources can protect from infectious diseases. This can be attributed to provitamin A potential of beta‐carotene (Elefson et al. 2023). Recent studies have proven that vitamin A enhances immunity and is an important anti‐inflammatory agent. In acute infectious diseases, it is reported that high levels of beta‐carotene can reduce CRP levels, a biomarker of inflammation. It does so by inhibiting the hepatic production of CRP. High blood concentrations of beta‐carotene have correlated with a lower risk of respiratory infections in advanced age (Promwong et al. 2023).

### 12.5 Diabetes Mellitus

Diabetes is a leading chronic disease that is prevailing worldwide. American Diabetes Association reported that by 2025, around 300 million will suffer from this disease. Free radical concentration, oxidative damage, and lipid peroxidation are involved in the pathogenesis of diabetes mellitus. Diets rich in glycemic load increase blood glucose levels, which leads to greater oxidative response, production of ROS, and cellular damage (Moitzi and König 2023). Kulawik, Cielecka‐Piontek, and Zalewski's (2023) investigation found that lycopene and beta‐carotene, among other phenolic compounds, lower hepatic aminotransferase, a biomarker for inflammation, when present in high serum levels. Lycopene is most efficient against this enzyme and thus protects from hyperglycemia. Moreover, when the blood glucose levels are significantly high, free radical production increases, and so does oxidative damage. This disturbs cellular homeostasis, leading to mitochondrial and DNA damage and other structures. The cell eventually goes through programmed death. Lycopene can lower glucose levels by modulating metabolism involving fats and glucose. Obesity and being overweight mean increased adipocytes, leading to an inflammatory response from inflammatory cytokines and chemokines. This inflammatory response plays a role in diabetes mellitus type 2. Lycopene can be stored in adipocytes due to lipid affinity, thus reducing DM and obesity (Geng et al. 2023).

A study by Jian et al. focused on the effect of lycopene supplementation on rats for a month. Overall glucose concentration was reduced due to a rise in insulin levels, and consequently, hyperglycemia was also managed at high doses (Kulawik, Cielecka‐Piontek, and Zalewski 2023). Diabetes also leads to multi‐ranging complications. Lycopene protects against microvascular complications, including pain associated with hyperalgesia. Lycopene also seemed to overturn this complication partly. Like lycopene, beta‐carotene has also been reported to act in obesity, fat metabolism and breakdown, insulin resistance, and diabetes mellitus type 2 (Kaur et al. 2023).

Beta‐carotene supplements ameliorate obesity‐related inflammation and complications; thus, it is also beneficial for type 2 diabetes mellitus, the outcome disease of obesity. High blood concentrations of beta‐carotene lowered insulin resistance in the subjects. Beta‐carotene levels were influenced by smoking or alcohol intake, which worsened insulin resistance in the first place (Ebadi et al. 2023; Aziz et al. 2023). Another research showed similar conclusions that patients with a habit of smoking had lower serum beta‐carotene content, approximately half than non‐smokers. This led to more insulin resistance and greater blood concentrations of glucose in smokers (Zheng et al. 2023). A study by Thomas, Calle, and Fernandez (2023) and Thomas, Ramkumar, et al. (2023) revealed that individuals who consumed a plant‐based diet rich in carotenoids had high levels of beta‐carotene in their blood, and their insulin resistance was also reduced. They proved that beta‐carotene protects pancreatic tissue against oxidative damage. Beta‐carotene acts as a metabolic modulator for fats and glucose. It regulates pancreatic functions and manages hyperglycemia. Beta‐carotene is an antioxidant and anti‐inflammatory agent that modulates insulin synthesis and release. Thus, it also counterchecks insulin resistance.

#### 12.5.1 Neurodegenerative Diseases

In developed countries, neurological diseases are on the rise. Parkinson's and Alzheimer's diseases are the most prevalent of them all and occur in 60% of these patients. Excessive free radical damage, inflammation, injury, and environmental and hereditary factors play a significant part in the etiology (Babazadeh et al. 2023). These factors lead to progressive damage that worsens over time, causing irreversible neuronal death. Neuronal damage disturbs nerve impulse transmissions, affecting the whole body, especially brain functioning. Neuronal membranes contain a rich amount of lipids in their structure, which makes them highly susceptible to lipid peroxidation (Ugbaja et al. 2024). Moreover, neurons cannot regenerate or reverse the damage. Thus, oxidation causes permanent free radical damage and function alteration in these cells. Carotenoids are free radical scavengers and are lipophilic. Lycopene has high antioxidant potential. It readily crosses the blood–brain barrier and can prevent further damage and inflammation in the brain, thus reducing the prognosis of neurodegenerative diseases (Kapoor et al. 2023).

Protein aggregation and folding of beta‐amyloid, alpha‐synuclein, and tau protein are the key etiological mechanisms in neurodegenerative diseases like AD and PD. These proteins alter organelles functioning, especially mitochondrial functioning. Lycopene is reported to reduce oxidative damage free radical production and toxic effects caused by the deposition and accumulation of these toxic proteins in the nervous system. Research on mice with PD induction suggested that lycopene intake can overturn neurodegenerative motor disorders in these subjects (Macar et al. 2023).

Plascencia‐Villa and Perry (2023) conducted a study that demonstrated that neuronal programmed death due to oxidative damage, misfolded protein‐related neurotoxicity, and mitochondrial dysfunction was halted by lycopene intake. Alzheimer's is an irreversible disorder, and no effective therapeutic options are available for reversal. Lycopene can be used in supplemental form for its antioxidant and neuronal protection activities as a preventive measure for Alzheimer's. Lycopene has also been reported to be effective in Huntington's disease (HD), which is a progressive genetic brain disorder. Lycopene prevents motor dysfunction and cognitive impairment in HD patients. Additionally, various studies have demonstrated that lycopene from dietary intake can inhibit inflammatory response in neuronal cells by downregulating NF‐kB activity. It also reduces cognitive impairment and depressive disorders (Ugbaja et al. 2024).

Research conducted by Abd Al Haleem, Ahmed, and El‐Naga (2023) on murine has demonstrated the neuroprotective potential of lycopene intake as it reduces oxidative damage of dopaminergic neuronal cells and restraining the reduction in dopamine. Moreover, lycopene intake for a long duration has been reported to decrease neuronal cell death and infarct volume or lesions in cerebrovascular ischemic patients and reduce the chances of leading to stroke. Beta‐carotene, similar to lycopene, prevents oxidative damage to neural tissues by reducing free radicals, but it is less efficient than lycopene. Beta‐carotene reduces ROS by enhancing concentrations of enzymes that catalyze these radicals. It can lead to overturning of cognitive dysfunction in AD. Beta‐carotene was also shown to reduce the accumulation of neurotoxic misfolded proteins in mice, improving brain functionality (Feng et al. 2023).

Beta‐carotene can also improve memory by binding with cholinergic enzymes such as AChE. Hence, it can also be used along with other therapies for AD. Beta‐carotene can also prevent DNA damage when consumed at low doses (Giap, Varatharajan, and Muthuraman 2023). A recent paper on Parkinson's reported that blood concentrations of carotenoids like beta‐carotene and lycopene were reduced in individuals with Parkinson's. Hence, their intake can reverse the risk to some extent risk (Kim et al. 2017).

#### 12.5.2 Skeletal System Disease

Osteoporosis is the most prevalent skeletal disease that affects bone by reducing its density and strength, making it weak and brittle. Bone mass decreases over time due to increased osteoclast activity and decreased osteoblast activity, leading to imbalance and porosity (Sobh et al. 2022). It affects mostly aged females, especially in postmenopausal times, as estrogen production decreases significantly. Age‐related increase in free radical damage is reported to enhance the prognosis of skeletal diseases. Oxidative damage reduces osteoblastic synthesis and functioning, leading to bone remodeling dysfunction. Increased ROS in the body enhances the activity of osteoclasts and the breakdown of osteocytes (Ru and Wang 2020). A study by Przybylska (2020) demonstrated that lycopene intake from food sources reduces ROS damage, thus reducing the ill effects of free radicals on osteocytes and preventing osteoporosis. Lycopene also can halt the production of bone resorption cells known as osteoclasts. Moreover, lycopene also prevents the maturation of these osteoclasts and the resorption of important minerals. Lycopene also shows potential in enhancing osteoblast reproduction by reducing cellular death and favoring the maturation of these cells. Wang, Heng, Song, et al. (2023), Wang, Li, Duan, et al. (2023), Wang, Lin, Liu, et al. (2023), Wang, Shen, Li, et al. (2023), Wang, Xu, Tan, et al. (2023), Wang, Zhang, Yin, et al. (2023), Wang, Zhang, Zhang, et al. (2023), and Wang, Zhou, Zheng, et al. (2023) study on murine in which the bone cells were exposed to ROS and later subjected to lycopene in high levels demonstrated that lycopene favors the production of bone‐forming cells and enhanced mineral uptake of bones leading to bone growth.

Walallawita et al. (2020) study on osteoporosis has proved the positive impacts of lycopene ingestion on bone mass, leading to lower recurrence of femoral fracture in postmenopausal women and osteoporosis‐related fractures in aged patients. Everyday ingestion of lycopene can decrease the chances of bone breakdown and remodeling in these aged women by preventing free radical damage to bone tissues. Beta‐carotene, on the other hand, reduced the survival of bone marrow‐derived macrophages and the production of osteoclasts, thereby downregulating pits production. Beta‐carotene can be used as a powerful medicine for severe osteoporosis and a supplement for mere prevention. Provitamin A activity of this carotenoid helps in retinoic acid receptor signaling, thus regulating bone metabolism. Zia‐Ul‐Haq, Riaz, and Modhi (2021) study demonstrated that beta‐carotene consumption increases bone mass by reducing the biomarker *T*‐score in postmenopausal females. Another study by Selvam and Rapeta (2018) revealed that blood concentrations of beta‐carotene were reduced in people with bone spurs. Thus, low beta‐carotene concentrations act as a significant determinant of this disease. These spurs can be inhibited by consuming sufficient beta‐carotene.

#### 12.5.3 Liver Diseases

The liver is the main organ for metabolizing and storing carotenoids after they are absorbed in GIT. Carotenoids exhibit their health‐related potential in this organ. Therefore, lycopene and beta‐carotene show their free radical scavenging abilities in preventing various liver‐related disorders (Clugston 2020). Lycopene exhibits the greatest protection and treatment potential in non‐alcoholic fatty liver disease (NAFLD). It has also been shown to protect liver cells against hepatitis‐related damage by restoring enzyme production and positively influencing lipoprotein metabolism (Lee et al. 2019).

Wang et al. researched to examine the hepatoprotective effects of lycopene. They reported that in non‐alcoholic steatohepatitis, lycopene reduces free radical damage and can prevent the development of cancer in hepatic cells due to the progression of NASH (Ibrahim et al. 2022). Another study proved that lycopene prevented NASH in animals by reducing ROS production. Consumption of beta‐carotene along with a healthy diet is beneficial for protection against liver diseases, especially NAFLD. Lycopene also reduces inflammation in the liver due to drug‐related damage. It maintains oxidative balance in the body (Ni et al. 2020). A study by Hedayati et al. (2019) proved that lycopene can ameliorate hepatic damage due to toxins and carcinogens by increasing antioxidant activities and liver detoxification with nuclear factor erythroid 2–related factor 2. Beta‐carotenoids exhibit provitamin A activity and scavenge free radicals, thus preventing oxidative damage in the body. Various new studies have implicated potential positive health impacts of beta‐carotene against free radical damage, inflammatory response, cellular death, fatty liver disease, and fibrotic scarring.

Balaji and Roy (2020) reported that beta‐carotene also prevents toxins and carcinogen‐related free radical damage, thus protecting against cancer development. Beta‐carotene supplementation effectively suppresses the toxic effects of aflatoxins by increasing serum concentrations of ascorbic acid and glutathione, which act as antioxidants. Mice‐based research in which they were fed beta‐carotene supplements and fatty meals demonstrated a reduction in serum cholesterol and AIP, lower fat buildup, and associated hepatic inflammatory response. It can be attributed to the inflammatory mediator's regulation of beta‐carotene. Liu et al. implied that beta‐carotene benefits hepatic steatosis by reducing HCV proliferation. Beta‐carotene scavenges free radicals and is a precursor of vitamin A; both of these characteristics make it beneficial for managing hepatitis, and it also protects from hepatic carcinogenesis (Ihnatowicz, Gębski, and Drywień 2023). Rich sources of beta‐carotene are effective against free radical damage, inflammation, and fibrosis in non‐alcoholic steatohepatitis in animals. These effects can be attributed to the modulatory activity of beta‐carotene in the inflammatory pathway and cellular death (Chen et al. 2021). Furthermore, high consumption of beta‐carotene or vitamin A rich foods is reported to be linked with reduced likelihood of hepatic carcinogenesis (Ebadi et al. 2023).

#### 12.5.4 Eye Diseases

Carotenoid intake has been linked with two eye‐related diseases, which are macular degeneration and cataract formation. Macular degeneration leads to central loss of vision as the macula is the central yellow part of the retina. This disease also leads to the formation of yellow spots in the vision (Mrowicka et al. 2022). Central vision loss often results in irreparable blindness in the aged population. A cataract is the cloudiness of the lens area that impairs vision. Cataract usually occurs in aged people and need to be extracted surgically. The exact pathogenesis of these two diseases is unknown, but free radical damage seems to be involved. Cataracts develop due to oxidative damage of eye proteins, leading to cluster formation and precipitation around the lens (Walchuk and Suh 2020). In an experiment, low blood levels of beta‐carotene have been associated with more susceptibility to macular degenerative diseases. Lycopene is also proven beneficial for people with compromised immunity and microvascular disorders related to brain damage (Johra et al. 2020). High blood concentrations of lycopene can reduce susceptibility to age‐related macular degeneration as lycopene can scavenge singlet oxygen ROS and lower plaque buildup in the eye, impacting the macula. Carotenoid consumption has been associated with multiple forms of cataract formation, enhancing the body's defense against this disease (Kamal et al. 2021). Studies report that beta‐carotene acts as an anticataract agent in people who consume a fruit‐ and vegetable‐rich diet. High blood concentration of this carotenoid reduces cataract risk as it acts as a free radical scavenger even at low oxygen pressure without causing any side effects (Choo et al. 2022).

## 13 Conclusion

Lycopene and beta‐carotene are molecules that belong to the class of carotenoids. These carotenoids confer promising prevention and help manage diseases like cancer, CVDs, DM, ocular, skin, oral, neurodegenerative, liver diseases, male infertility, and inflammatory disorders. These properties have been associated with these compounds' antioxidant potential and unique physiochemical structures. However, they can perform their role in disease prevention and therapy with a balanced diet. Consumption of fruits and vegetables containing (lycopene and beta‐carotene) should be encouraged for health promotion and disease protection. However, more research is needed to unveil the proper mechanisms of actions of these compounds in various disorders with appropriate emphasis on the exact dose.

## Author Contributions

**Tabussam Tufail:** conceptualization (equal), formal analysis (equal). **Huma Bader Ul Ain:** investigation (equal), methodology (equal). **Sana Noreen:** supervision (equal), validation (equal). **Ali Ikram:** writing – original draft (equal), writing – review and editing (equal). **Muhammad Tayyab Arshad:** data curation (equal), validation (equal). **Muhammed Adem Abdullahi:** investigation (equal), supervision (equal).

## Ethics Statement

This study did not involve humans or animals.

## Consent

This study did not involve humans.

## Conflicts of Interest

The authors declare no conflicts of interest.

## Data Availability

The data that support the findings of this study are available on request from the corresponding author. The data are not publicly available due to privacy or ethical restrictions.
